# A systematic review of hepatitis B screening economic evaluations in low- and middle-income countries

**DOI:** 10.1186/s12889-018-5261-8

**Published:** 2018-03-20

**Authors:** Cameron M. Wright, Lydia Boudarène, Ninh Thi Ha, Olivia Wu, Neil Hawkins

**Affiliations:** 1 Health Systems and Health Economics, School of Public Health, Faculty of Health Sciences, Curtin University, Perth, Western Australia Australia; 2 School of Medicine, Faculty of Health, University of Tasmania, Hobart, Tasmania Australia; 3 Faculty of Public Health and Policy, London School of Hygiene and Tropical Medicine, London, UK; 4 Institute of Health and Wellbeing, University of Glasgow, Glasgow, Scotland UK

**Keywords:** Costs and cost analysis, Hepatitis B, Hepatitis, viral, human, Economics, medical

## Abstract

**Background:**

Chronic hepatitis B infection is a significant cause of morbidity and mortality worldwide; low- and middle-income countries (LMICs) are disproportionately affected. Economic evaluations are a useful decision tool to assess costs versus benefits of hepatitis B virus (HBV) screening. No published study reviewing economic evaluations of HBV screening in LMICs has been undertaken to date.

**Methods:**

The following databases were searched from inception to 21 April 2017: MEDLINE, PubMed, EMBASE, CINAHL Plus, the Cochrane Library, Global Health and the Cost-effectiveness Analysis Registry. English-language studies were included if they assessed the costs against the benefits of HBV screening in LMICs. PROSPERO registration: CRD42015024391, 20 July 2015.

**Results:**

Nine studies fulfilled the eligibility criteria. One study from Thailand indicated that adding hepatitis B immunoglobulin (HBIG) to HBV vaccination for newborns following screening of pregnant women might be cost-effective for some LMICs, though inadequate total funding and health infrastructure were likely to limit feasibility. A similar study from China indicated a benefit to cost ratio of 2.7 from selective HBIG administration to newborns, if benefits were considered from a societal perspective. Of the two studies assessing screening amongst the general adult population, a single cost-benefit analysis from China found a benefit to cost ratio (BCR) of 1.73 with vaccination guided by HBV screening of adults aged 21–39, compared to 1.42 with vaccination with no screening, both from a societal perspective. Community-based screening of adults in The Gambia with linkage to treatment yielded an incremental cost per disability-adjusted life year averted of $566 (in 2017 USD), less than two-times gross domestic product per capita for that country.

**Conclusions:**

Screening with ‘catch-up’ vaccination for younger adults yielded benefits above costs, and screening linked with treatment has shown cost-effectiveness that may be affordable for some LMICs. However, interpretation needs to account for total cost implications and further research in LMICs is warranted as there were only nine included studies and evidence from high-income countries is not always directly applicable.

**Electronic supplementary material:**

The online version of this article (10.1186/s12889-018-5261-8) contains supplementary material, which is available to authorized users.

## Background

The hepatitis B virus (HBV) can lead to acute and/or chronic hepatitis B infection (CHB). HBV can be transmitted vertically, horizontally (e.g. child-to-child), sexually or parenterally [1]. Hepatitis B surface antigen (HBsAg)-positive individuals who are also positive for the hepatitis B e antigen (HBeAg) are at an especially high risk of transmitting HBV to others [2]. For women who are HBeAg-positive, administering hepatitis B immunoglobulin (HBIG) to newborns, as an addition to HBV vaccination can further prevent the risk of vertical transmission [3]. The vast majority of HBV-related deaths are due to longer-term complications from CHB, defined as HBsAg persistence for  more than six months. These include cirrhosis and hepatocellular carcinoma (HCC) [2]. An estimated 686,000 people died due to HBV infection in 2013 [4]. The highest prevalence of HBsAg persistence is in sub-Saharan Africa, East Asia, the Amazon and southern part of eastern and central Europe [5]. The complications of CHB are costly to manage, the authors of a 2009 study from China [6] report annual per-person costs of between $1636 and $6054. Although HBV vaccination has decreased incidence, prevalence of HBV in the low and middle-income countries (LMICs) remains high due to insufficient coverage rates of vaccination and limitations in other preventive measures [7].

Screening for HBV, usually via HBsAg (see Table 1), can help identify individuals at risk of developing complications from HBV infection and/or of transmitting it to others. This is important, as people infected with HBV may well be unaware [8]. With advances in point-of-care testing, screening for HBV is convenient and inexpensive [9]. The cost of treatment has traditionally been a barrier restricting follow-up treatment, though this cost has decreased in recent years [10, 11]. International guidelines recommend ‘high-risk’ groups screening (e.g. household and/or sexual contacts of persons with CHB) [3, 12]. However, the feasibility of screening in many LMICs is limited, including of pregnant women to reduce vertical transmission [13], amongst whom screening uptake in LMICs is low [14]. Given a high prevalence of HBV in LMICs, relative to high-income countries (HICs), screening of the general population rather than targeting specific subpopulations may be a more effective strategy to reduce the HBV burden. The World Health Organization (WHO) conditionally recommends general population testing for settings with HbsAg seroprevalence of ≥2%, and strongly recommends screening of pregnant women in settings with the same seroprevalence ([12], p. xxviii). The cost-effectiveness of screening is an important consideration, especially in resource-constrained settings [15]. An understanding of the economic evidence available to guide HBV screening policy in LMICs is therefore important.

**Table 1:** Interpretation of markers used in hepatitis B screening [2, 53]

Marker (abbreviation)	Description
Hepatitis B surface antigen (HBsAg)	marker of acute or chronic hepatitis B infection
Hepatitis B surface antibody (anti-HBs)	a high level indicates previous infection or response to vaccination and current immunity (generally ≥10 IU/L considered ‘protected’)
Hepatitis B e antigen (HBeAg)	presence indicates high infectivity
Total hepatitis B core antibody (anti-HBc)	indicates resolved infection if positive for this and hepatitis B surface antibody, but negative for HBsAg; will be positive along with HBsAg in acute or chronic hepatitis B infection; if positive but negative for HBsAg and hepatitis B surface antibody, usually indicates distant resolved hepatitis B infection

To date, there appears to be no published review assessing economic evaluations of HBV screening in LMICs. Because there are differences in HBV epidemiology and in the application of cost-effectiveness thresholds in LMICs, and because HBV is disproportionately concentrated in these countries, a critical review of studies assessing HBV screening cost-effectiveness in LMICS is indicated to guide policy and research in this area. This study aimed to: i) systematically review the available economic evidence on HBV screening of the general population/specific sub-populations in LMICs and provide a narrative synthesis of progress so far, and; (ii) analyse the strengths and limitations of existing economic evaluations, to provide research and policy recommendations for future research.

## Methods

### Protocol registration and reporting structure

A protocol for this review was prospectively registered on 20 July 2015 (PROSPERO Registration: CRD42015024391 [16]. This report was based on the Preferred Reporting Items for Systematic Reviews and Meta-Analyses statement [17].

### Eligibility criteria

A priori inclusion criteria for this review were categorised according to the population, intervention, comparator, outcome and setting (PICOS) format.

### Inclusion criteria

**Population:** the general population (including blood donors, but not if screening is not linked to follow-up treatment), or any specified subpopulation.

**Intervention:** screening for HBV.

**Comparator:** no screening (e.g. universal vaccination) or alternate screening scenarios.

**Outcome:** measuring and reporting quantitative costs and benefits.

**Setting:** studies conducted in/using data from LMICs as defined for the 2016 fiscal year by the World Bank and/or using data from these countries [18].

### Exclusion criteria

1. Studies not considered ‘full economic evaluations’ (i.e. considering either costs *or* consequences of HBV screening, but not both in relation to one another). Studies assessing the HBV prevalence at which screening becomes less expensive than universal HBV vaccination (i.e. cost-minimisation analyses), systematic reviews and conference abstracts were also excluded.
2. Studies with no full-text version available in English.

### Information sources

Two systematic reviews on HBV screening economic evaluations had already been conducted at the time this project was commenced, though the eligibility criteria meant that only studies from HICs were included [19, 20]. The following databases were searched from inception to 2 August 2015: MEDLINE (via OVID), PubMed, EMBASE (via OVID), the Cumulative Index to Nursing and Allied Health Literature (CINAHL) Plus (via EBSCO), the Cochrane Library, EconLit (via OVID), Global Health (via OVID), Open Grey and the Cost-effectiveness Analysis (CEA) Registry [21]. An update of the search, excluding EconLit and Open Grey, was conducted on 21 April 2017.

Search strategies were based around the National Health System Economic Evaluation Database (NHS EED) filters available for MEDLINE, PubMed, EMBASE and CINAHL [22]. The search for each database roughly comprised: <terms from NHS EED filter to identify economic evaluation, removing date, article type and journal restrictions> AND < terms to identify studies on HBV in humans> AND < terms to identify studies on screening>. Medical Subject Heading and EMTREE (for EMBASE) terms were used where appropriate, in addition to free text terms with relevant truncations (e.g. test$), using or adapting search terms reported by Geue and colleagues [20]. The search strategies used for each database are provided in Additional file 1.

Experts at the London School of Hygiene and Tropical Medicine (and elsewhere, as referred) were contacted in June 2015 to identify unpublished work that may be missed through database searching. Unduplicated references were checked by hand for each included paper, as were the references for the screening section of recently published WHO HBV guidelines [3]. The Web of Science database was searched on 14 July 2017 for studies citing included articles.

### Study selection

The title and abstract of unduplicated articles were screened against each eligibility criterion in-turn by one author (CMW or NTH). Two authors (CMW, LB and/or NTH) independently screened articles advancing to full-text review, with disagreements resolved through discussion. The reason for article rejection was recorded at both screening stages.

### Data collection process and items

The Consolidated Health Economics Evaluation Reporting Standards (CHEERS) statement was used as a basis for the data extraction form [23]. Data were grouped into the areas of: 1) title and abstract; 2) introduction; 3) methods; 4) results; 5) discussion; and 6) other (refer to [23] for specific data items). Data were extracted in the currency used in the study and, if necessary, converted to United States Dollars (USD) using the currency conversion figure provided in the paper or, if not reported, historical conversion rates for 30 June of the year of publication [24]. For this review reported costs were expressed in 2017 USD (inflated from mid-year using consumer price indices [25]). Two authors extracted data independently (shared between CMW, LB and NTH). Disagreements in data extraction were settled through discussion.

### Critical appraisal

The Consensus of Health Economic Criteria (CHEC) checklist and the quality appraisal tool developed by Philips and colleagues were used to critically appraise included studies [26, 27]. Where relevant, the Drummond et al. checklist was also consulted [28]. As this review focused on LMICs, the methodological specifications of the Gates Reference Case were used to assess the appropriateness of study methods [29].

## Results

### Study selection

**Fig. 1:**
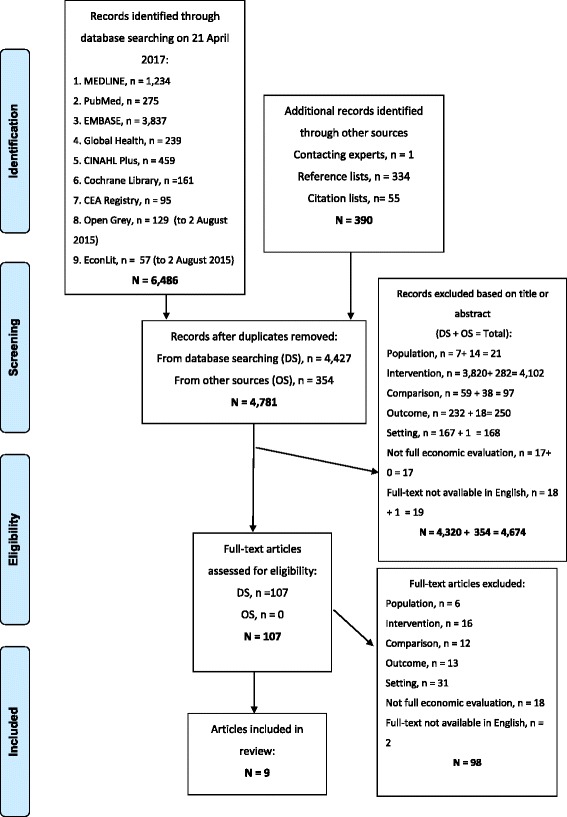
Flow diagram of study selection

Database searching returned 4427 unduplicated records. Of these, 4320 were removed during title and abstract screening, leaving 107 articles to undergo full-text review. Nine of these articles fulfilled the eligibility criteria [30–38]. A further 354 unduplicated articles were identified via reference and citation checking; none of these fulfilled the eligibility criteria. Figure 1 shows the search strategy results in detail; rejected articles at the full text review stage are listed in Additional file 2.

### Study characteristics

The included studies were published from 1989 through 2016 and used data from Iran [30], India [31], South Africa [33], the Philippines [34], Thailand [36, 37], China [32, 38] and The Gambia [35]. More recent studies were conducted and reported with greater adherence to contemporary economic evaluation guidelines (see ‘critical appraisal’ section in methods). Five studies focused on screening strategies for pregnant women and the resulting clinical intervention that would then be appropriate for the infant postpartum [31–34, 36]. The remaining studies focused on screening adults [30, 35, 37, 38]. Studies reported variable outcomes. Summary characteristics and main results for included studies are provided in Table 2, with the complete data extracted from each study provided in Additional file 3.

**Table 2:** Summary of included studies, with further details in Additional file 3. ‘Infection’ refers to chronic hepatitis B infection

Study author(s) and year of publication, listed by subpopulation targeted, in reverse chronological order	Setting and population	Study design	Strategies compared	Main results (costs in 2017 United States Dollars)^a^
Antenatal screening				
Chen et al., 2016 [32]	China, pregnant women	Decision tree (for outcome post-intervention), linked with Markov model (for health outcomes, if infected with HBC).	(1) no screening, no vaccination or (2) no screening, universal three-dose hepatisis B vaccination (HBV) vaccination for newborns both compared to (3) screening, universal three-dose HBV vaccination + hepatitis B immunoglobulin (HBIG) for newborns of hepatitis B antigen surface antigen (HBsAg)-positive.	(3) versus (1): 12.49 million infections and 0.58 million early deaths averted. Direct and societal costs of averted illness was $12.25 billion and $47.35 billion, respectively. Benefit to cost ratios (BCRs) from direct and social perspective of 61.3 and 193.2, respectively. Sensitivity analyses indicated BCRs remaining above 1.0 regardless of changes in parameter values. (3) versus (2): there were 3500 infections averted. BCRs from direct and social perspective of 0.4 and 2.7, respectively. No costs averted were reported.
Vimolket and Poovorawan, 2005 [36]	Thailand, pregnant women	Decision tree modelling	(1) Universal vaccination of newborns, HBIG if mother HBsAg-positive versus (2) universal vaccination of newborns, HBeAg if HBsAg-positive, HBIG if positive for both versus (3) universal vaccination of newborns, no screening, no HBIG (current strategy) versus (4) no vaccination of newborns, no screening of pregnant women.	(1) Cost = $944, expected cases prevented = 99.9, cost-effectiveness (CE) = $9.5 baht/case prevented, Incremental cost-effectiveness ratio (ICER) = $3067 /case prevented, relative to strategy 2). Total annual cost (for 800,000 cohort) = $7.55 million. (2) Cost = $852, expected cases prevented = 99.87, CE = $8.5/case prevented, ICER = $646 per case prevented, relative to strategy 3). Total annual cost = $6.82 million. (3) Cost = $484, expected cases prevented = 99.30, CE = $4.9/case prevented, ICER = $211/case prevented, relative to strategy 4). Total annual cost = $3.87 million (4) Cost = $0, expected cases prevented = 97, CE = 0, ICER N/A. Through strategy 4 cases are still ‘prevented’ (newborns are born without HBV infection) because model assumes only 3% of newborns will become a carrier via vertical transmission for this population.
Aggarwal and Naik, 1994 [31]	India, pregnant women	Decision tree modelling	(1) Universal vaccination, no screening versus (2) HBsAg screening, vaccination for newborns only if mother positive.	(1) 341.2 carriers/10,000 newborns born prevented, total cost $71,169, therefore $209/carrier prevented. (2) 44.8 carriers/10,000 newborns born prevented, total cost $36, 713, therefore $819/carrier prevented
Guidozzi et al., 1993 [33]	South Africa, pregnant women	Prevalence study in relation to a ‘hypothetical worst-case scenario’	(1) Screening and HBV vaccination of newborns if mother HBsAg-positive versus (2) no screening, no vaccination.	Estimation of 7 HBV infections averted (1/500 births; 3469 women screened), total cost of $44,029, costs per case averted (compared to no screening) of $6290 with cost of each HBsAg screening test estimated as $12.7.
Lansang et al., 1989 [34]	Philippines, pregnant women	Decision tree modelling	Three screening strategies ( (1–3) rapid ‘finger prick’ test, venous sampling with two different assays) with: a) selective HBV vaccination of newborns +/− b) HBIG and (4) universal vaccination.	Cost-effectiveness defined as expected cost (EC in pesos)/expected ‘utility’^b^ (EU) per person) (1) a) HBV vaccine alone: EC = $1.2, EU = 0.9, EC/EU = $1.3 b) HBV vaccine & HBIG: EC = $3.3, EU = 0.9, EC/EU = $3.6 (2) a) HBV vaccine alone: EC = $1.2, EU = 0.89, EC/EU = $1.4 b) HBV vaccine & HBIG: EC = $2.3, EU = 0.89, EC/EU = $2.6 (3) a) HBV vaccine alone: EC = $9.2, EU = 0.90, EC/EU = $10.2 b) HBV vaccine & HBIG: EC = $9.8, EU = 0.9, EC/EU = $11 (4) EC = $14.8, EU = 0.95, EC/EU = $15.6
Screening of adults				
Nayagam. et al., 2016 [35]	The Gambia, adults 38 years old or older	Decision tree combined with Markov models	Comparing screening and treatment versus current practice of no publicly provided screening or treatment.	498 additional disability-adjusted life years (DALYs) averted, 417 life year (LY) gained, or 526 quality-adjusted life years (QALYs) saved (all per round). Incremental cost-effectiveness ratios (ICERs) of $566/ DALY averted, $677 /LY saved, $536 / QALY gained
Zheng et al., 2015 [38]	China, adults	Decision tree modelling	Adults separately analysed as 21–39 year olds, 40–59 year olds, and 21–59 year olds together comparing: (1) Vaccination of adults with no screening or (2) vaccination of adults following screening for HBV core antigen both compared to (3) no vaccination, no screening of adults.	Young adults (21–39 years): (1) versus (3): direct costs of $1.54 billion and costs averted $1.64 billion; societal costs of $2.16 billion and costs averted of $3.08 billion, yielding BCRs of 1.06 and 1.42 respectively (2) versus (3): direct costs of $1.64 billion and costs averted $1.95 billion; societal costs of $2.16 billion and costs averted of $3.70 billion, yielding a BCRs of 1.19 and 1.73 respectively. Middle-aged adults (40–59 years): (1) versus (3): direct costs of $1.44 billion and costs averted of $0.82 billion; societal costs of $2.06 billion and costs averted of $1.23 billion, yielding BCRs of 0.59 for both perspectives; (2) versus (3): direct costs of $1.54 billion and costs averted of $1.03 billion; societal costs of $2.06 billion and costs averted of $1.54 billion, yielding BCRs of 0.68 and 0.73 respectively.
Wiwanitkit, 2009 [37]	Thailand, people travelling abroad for work	Extrapolation of cross-sectional study data to 10,000 person cohort	(1) Screening before travelling abroad versus (2) returning home if found to be infected with HBV after travelling abroad for work.	(1) Cost of screening 10,000 workers = $75, 711 (2) Cost of returning home from within Asia (if no workers screened prior to leaving) = $157,479. Cost from returning home from outside of Asia (if no workers screened prior to leaving) = $472,437. Therefore, cost saving from screening 10,000 workers prior to leaving = $81,768 – $396,726
Adibi et al., 2004 [30]	Iran, pre-marriage couples	Decision tree modelling	(1) Screening for HBsAg and giving protection protocol (HBV vaccine, condoms to seronegative partner, if other individual is seropositive or (2) as above with further screening for HBV core antibody and protection protocol if negative for both (and partner positive) both compared to (3) no screening or prevention.	(1) versus (3): $ 269/infection averted (2) versus (3): $ 263/infection averted Threshold analysis (discounted at 3%, cost of CLD above which screening strategies are cost saving): (1) versus (3): $3758 (2) versus (3): $ 3663

Abbreviations: *BCR* benefit to cost ratios, *CE* cost-effectiveness, *DALY* disability adjusted life year, *EC* expected cost, *EU* expected utility, *HBsAg* hepatitis B surface antigen, *HBIG* hepatitis B immunoglobulin, *HBV* hepatitis B vaccine, *ICER* incremental cost-effectiveness ratio, *QALY* quality adjusted life year

^a^Where costs in the original study were expressed in another currency, these data were converted to United States Dollars (USD) using the conversion figure in the paper or, if not available, historical conversion rates for June 30 of the year of publication [24], and then inflated to 2017 USD using consumer price indices [25]. Note that due to different study designs and study periods, even though costs are expressed in 2017 USD, this does not mean costs are directly comparable between studies

^b^Utility = one minus the probability of HBV infection through each compared strategy

### Study findings

### Antenatal screening

Vimolket and Poovorawan [36] analysed the addition of HBIG to a universal 3-dose HBV vaccination programme for newborns in Thailand, following HBV screening of pregnant women. The incremental cost-effectiveness ratio (ICER), expressed as cost per case prevented, was $211 for universal vaccination relative to the next least costly option of no vaccination of newborns. This was much less than the $3067 ICER for screening of HBsAg, followed by 3 doses of HBV vaccine over six months for the infant if the mother tests positive, compared to a 2-stage screening strategy universal HBV vaccination and HBsAg screening, followed by HBeAg if positive, with HBIG administered if HBeAg-positive. This in-turn had an ICER of $646 per case prevented, when compared to universal vaccination with no screening. These authors state that a challenge to interpreting their results is that, “there is no socially acceptable threshold for cost per case prevented to guide decisions…” [36]. They conclude that universal vaccination with no screening should continue with current programme funding levels, though a policy of HBsAg followed by HBeAg screening if positive, with HBIG administration if positive for both could be cost-effective and feasible if funding could be doubled [36].

More recently, Chen and colleagues [32] reported a cost-benefit analysis comparing HBV vaccination with HBIG administration for newborns of HBsAg-positive mothers to either, universal HBV vaccination or no vaccination, both with no screening. The ‘base case’ analysis compared HBV vaccination, with HBIG only for newborns of mothers screening positive for HBsAg, with no vaccination. This yielded a benefit to cost ratio (BCR) of 193, from societal perspective. BCRs for this comparison were insensitive to prices changes of HBV vaccine or HBIG. Compared to universal HBV vaccination, screening mothers with the addition of HBIG for newborns of HBsAg-positive mothers averted 3500 HBV cases (in a birth cohort of 16.4 million newborns) and yielded BCRs of 0.4 (direct costs) and 2.7 (from a societal perspective).

The other three older studies of antenatal screening [31, 33, 34] primarily assessed HBV screening as a means of targeting HBV vaccination, versus universal vaccination. Two of these studies, one each from South Africa and India, recommended universal HBV vaccination rather than screening-based vaccine targeting [31, 33], whilst the third study from the Philippines found screening most likely to be cost-effective when the cost of HBV vaccine was very high [34].

### Screening of pre-marital couples

The authors of the study from Iran report an economic analysis comparing strategies screening premarriage couples of HBV, via HBsAg (strategy 1) plus the core antibody for negative partners in serodiscordant couples (strategy 2) [30]. An average cost-effectiveness of $269 per chronic infection averted was reported for strategy 1; for strategy 2 the value was $263. The authors assessed the impact of the values they included in their model and found that uncertainty associated with these could give a range of $89 – $440 and $88 – $412 for strategy 1 and 2 respectively. The authors modelled the costs of screening a 25-year old, to a 50-year old developing chronic liver disease (CLD) secondary to CHB for ten years, discounting costs at 3%. They estimate that above a cost of $3758 for strategy 1 and $3663 for strategy 2 for managing CLD, screening of premarriage couples is likely to be cost saving. This study assumed no pre/extra-marital sex.

### Screening of people traveling abroad for work

One of the studies from Thailand modelled the costs of screening people travelling abroad for work for HBsAg prior to leaving, versus the cost of returning home to Thailand if HBV infection is detected once they are abroad [37]. The author estimates cost savings of $81,768 to $396,726 for a hypothetical cohort of 10,000 workers. This is a brief article, which does not explain why returning is necessary.

### Screening of general adult population

Authors of a recent study from China assessed the effect of providing 3 doses of HBV vaccination to adults aged 21–39 years, and 40–59 years [38]. They assessed the costs of vaccination with or without screening to determine who should be offered ‘catch up vaccination’, versus the costs averted from reduced HBV-related morbidity and mortality. The results of this cost-benefit analysis were given from both a direct costs and a societal costs perspective. Societal costs included indirect costs of earning potential work forfeit via HBV-associated morbidity and early mortality. For younger adults aged 21–39 years, the BCR was 1.19 considering direct costs, and 1.73 from a societal perspective (i.e.). This is compared to 1.06 and 1.42 respectively for vaccination of this age group without prior screening. The BCR remained > 1 for sensitivity analysis of model parameters including vaccine cost. The BCRs were less than 1 for adults aged 40–59 years, indicating the costs of the program would exceed the expected costs averted. The total costs exceeded $1.4 billion for each scenario. The authors recommended that policy makers consider a targeted HBV vaccination program for younger adults in China.

Authors of a recent study from The Gambia [35] modelled community-based screening of adults at mean age 38 with a rapid HBsAg test, followed by treatment with tenofovir where indicated. The base model in this analysis, where HbsAg positive prevalence was 8.8%, generated ICERs of $566 per disability-adjusted life year (DALY) averted, $677 per life year gained and $536 per quality-adjusted life year gained. In sensitivity analyses, the drug cost and treatment, cost of screening and progression of HBsAg-positive, but HBeAg-negative patients to decompensated cirrhosis had the greatest potential to increase the ICERs. With a willingness-to-pay of three-times the gross domestic product of The Gambia, screening was very likely to be cost-effective, though with a more conservative World Bank target of less than $240 per DALY averted, screening was unlikely to be cost-effective. Authors of this study recommended integration of screening into existing public health programs [35].

### Critical appraisal of included studies

All but three of the studies assumed 100% compliance with interventions [32, 35, 38]. The choice of decision tree modelling limited the ability to formally assess consequences over time for most studies [30, 31, 34, 36, 38]. However, the studies from China and The Gambia [32, 35, 38] took a lifetime perspective and considered morbidity and death resulting from CHB. Cost data primarily consisted of direct costs (e.g. of the screening itself). The perspective of the analysis (i.e. who was paying the costs) was stated clearly for only four of the studies [30, 35, 36, 38]. In the cases were costs and benefits were discounted, an appropriate value of 3% was used [29, 30, 32, 35, 38]. Clearly reported sensitivity analyses were performed for five studies [30, 32, 35, 36, 38]. Among studies for which sensitive analyses were reported, major influential parameters include costs of screening tests [35, 36], prevalence of HBV, screening uptake rate, health utility calculation and discount rate [35].

## Discussion

This review demonstrates that there are few economic evaluations of HBV screening in LMICs. More recent studies have most relevance to contemporary public health practice and were generally reported with greater transparency and consideration of the effect of uncertainty on the results generated. Earlier studies tended to focus more on vaccination, the cost-effectiveness of which in LMICs has been reviewed elsewhere [39, 40]. While the cost-effectiveness of HBV screening has been reviewed for studies conducted in HICs [19, 20], in LMICs there is relatively less concentration of HBV in ‘high risk’ groups and there are often markedly different willingness-to-pay thresholds [41].

Three studies included in this review evaluated the cost-effectiveness of adding HBIG to HBV vaccination for newborns born to HBsAg +/− HBeAg-positive mothers [32, 34, 36]. The outcomes reported and approach taken by the authors of 1989 study [34] limits the usefulness of their results. The authors of the more recent study by Vimolket and Poovorawan [36] conclude that feasibility in resource-constrained settings was limited by allocated budget and logistical support required to implement the service [36]. A more recent study from China, by Chen and colleagues [32] indicated a benefit above cost from a societal perspective for adding HBIG to HBV vaccination for newborns of HBsAg-positive mothers. However, the assessment of uncertainty in this modelling focused on the comparator with no vaccination. Given high HBV vaccine coverage in China [42], further exploration of the comparison with universal vaccination would have been helpful. Authors of a 2013 study assessed the cost-effectiveness of augmenting HBV vaccination with HBIG, this time with cost data from Taiwan, a HIC [43]. These authors model the ICERs of different HBV screening strategies over four levels of HBV prevalence 1%, 5%, 15% and 25%. However, each of the strategies explored by these authors, involving screening and administering HBIG based on the results, had incremental costs per infection averted, relative to universal vaccination without screening, >$1500 (in 2011 USD), likely limiting the cost-effectiveness in many LMICs.

With the increase in coverage of universal infant HBV vaccination, the horizontal transmission of HBV has decreased, with a reduction in HBsAg prevalence among children seen in many settings including China [44] and Cambodia [45]. With ~ 50% likelihood of progression to CHB if infected as a child, this should lead to gains in terms of lower CHB amongst vaccinated populations as they age [46]. However, the WHO estimates the full effects – via a reduction in HBV-associated mortality – of universal HBV vaccination of new-borns will not be realised for 20 to 40 years [3]. Treatment has been found to markedly reduce the progression of CHB to complications of cirrhosis and HCC [47]. The screening principles put forward by Wilson and Jungner [15] highlight the importance of having an “accepted treatment” and “agreed policy on whom to treat”. Recently published WHO guidelines thus strengthen the case for HBV screening [3, 12]. The authors of the study from China [38] sought to assess the BCR of ‘catch up’ three-dose HBV vaccination for young adults who may not have benefitted from universal infant vaccination, from a societal perspective. These authors report that screening prior to vaccination yielded a higher BCR amongst younger adults (21–39 years). Only one study assessed community-based screening linked to treatment and found the cost-effectiveness to be dependent on the willingness-to-pay threshold applied [35].

Given that DALY averted is used in the WHO’s CHOICE values [48], future studies should include this as one of the outcome measures (indicative disability weights for The Gambia have been published [39]). Considering the ICER in terms of total budget impact is also important, especially for applying the findings from these more widespread strategies in the studies from China and The Gambia [39, 49]. This is because willingness-to-pay thresholds differ between countries, and need to be applied with due consideration of context and competing health priorities [41]. More widely, Nayagam and colleagues [50] have separately modelled the costs of global elimination of HBV including a widespread testing and treating strategy.

Geue and colleagues [20] have critically assessed cost-effectiveness modelling studies of HBV screening in Organisation for Economic Cooperation and Development member countries and their findings of higher quality amongst more recent studies is consistent with our findings. A systematic review by Hahné and colleagues [19] also included cost-effectiveness studies from HICs. While these authors’ review focused on implications of HBV screening economic evaluations for European countries, some of the findings may have implications for future research in LMICs. They suggest considering that the cost-effectiveness of antenatal screening might be improved if antiviral treatment of the mother is also considered [19]. This is not a scenario explored by the antenatal screening studies from LMICs included in our review. Since database searching, a narrative review of ‘test and treat’ strategies as relevant to LMICs has been reported by Nayagam and colleagues [51]. Consistent with our findings, these authors were only able to find one relevant study conducted in LMICs [35]. Our study should be read in conjunction with these reviews, especially given the small number of studies conducted in LMICs.

Strengths of this study include a comprehensive search strategy, methodological critical appraisal and prospective protocol registration. Limitations of the review included exclusion of non-English language studies, of studies screening blood where no linkage to treatment followed, and that only nine studies fulfilled the eligibility criteria, meaning that any publication bias may have an impact on the conclusions drawn from the literature. The definition of LMICs for the 2016 fiscal year could have created issues where countries had changed from low-or middle-, to high-income. However, this was not an issue during study selection. That we have expressed costs in 2017 USD does not mean costs between studies are directly comparable, due to different study methods and periods. Finally, the different target populations, different prevalence of HBV infection (for example, between China [44, 52] and The Gambia [35]) and that the two studies looking at the general population took different approaches – one a cost-utility analysis [35], the other a cost-benefit analysis [38] – limited our ability to aggregate findings quantitatively across studies.

## Conclusions

Further, high quality study of HBV screening cost-effectiveness in LMICs is warranted, ideally linked with treatment for those found seropositive, as community-based screening has the potential to require significant investment. Current evidence is equivocal regarding the feasibility of screening of pregnant women linked with HBIG administration to newborns in LMICs, though the WHO recommends screening amongst this subpopulation ([12], p. xxviii). At this stage, screening with ‘catch-up’ vaccination for younger adults has some evidence of benefit above cost, though from a single study [38], whilst a study assessing screening people > 30 years, with follow-up treatment, has yielded ICERs that may be affordable for some LMICs [35].

## Additional files

Additional file 1: Database search strategies. (DOCX 20 kb)

Additional file 2: Rejected articles undergoing full-text review. (DOCX 27 kb)

Additional file 3: Data extracted from included studies. (DOCX 71 kb)

## Abbreviations

BCR: Benefit to cost ratio
CHB: Chronic hepatitis B
DALY: Disability-adjusted life year
HBeAg: Hepatitis B e antigen
HBIG: Hepatitis B immunoglobulin
HBsAg: Hepatitis B surface antigen
HBV: Hepatitis B
HCC: Hepatocellular carcinoma
HIC(s): High income country(ies)
ICER: Incremental cost-effectiveness ratio
LMIC(s): Low- or middle-income country(ies)
LY: Life year
PICOS: Population, intervention, comparator, outcome, setting
PRISMA: Preferred reporting items for systematic reviews and meta-analyses
QALY: Quality-adjusted life year
USD: United States Dollar
