# Supplementary material for: A systematic review of hepatitis B screening economic evaluations in low- and middle-income countries
Source: BMC Public Health. 2018 Mar 20;18:373. doi: 10.1186/s12889-018-5261-8 (PMC5859762; doi:10.1186/s12889-018-5261-8)
Supplement: Supplementary file 1 — Database search strategies. (DOCX 20 kb) [file 12889_2018_5261_MOESM1_ESM.docx]

## Additional file 1. Database search strategies

**MEDLINE search strategy**

1. economics/

2. exp "costs and cost analysis"/

3. economics, dental/

4. exp economics, hospital/

5. economics, medical/

6. economics, nursing/

7. economics, pharmaceutical/

8. (economic$ or cost or costs or costly or costing or price or prices or pricing or pharmacoeconomic$).ti,ab.

9. (expenditure$ not energy).ti,ab.

10. value for money.ti,ab.

11. budget$.ti,ab.

12. or/1-11

13. ((energy or oxygen) adj cost).ti,ab.

14. (metabolic adj cost).ti,ab.

15. ((energy or oxygen) adj expenditure).ti,ab.

16. or/13-15

17. 12 not 16

18. exp animals/ not humans/

19. 17 not 18

20. hepatitis, viral, human/

21. exp hepatitis b/

22. ((hepatitis or hep) adj b).ti,ab.

23. HBV.ti,ab.

24. 20 or 21 or 22 or 23

25. exp mass screening/

26. test$.mp.

27. case find$.mp.

28. screen$.mp.

29. 25 or 26 or 27 or 28

30. 19 and 24 and 29

**Explanatory notes:** Removed date and article type limits and terms relating to specific journals from NHS EED filter. Adding additional MeSH term ‘exp hepatitis b virus/’ (not used by Geue and colleagues [23]) yielded three additional hits that were clearly irrelevant to the review, therefore this term was not added to the search strategy. Additional terms for hepatitis and screening identical to Geue and colleagues as relevant MeSH terms were included and free-text terms were appropriate.

**PubMed search strategy**

((((((((economic evaluation*[ti]) OR economic analy*[ti]) OR cost analy*[ti]) OR cost effectiveness[ti]) OR cost benefit*[ti]) OR cost utilit*[ti])) AND ((((hepatitis) OR hepatitis b[tiab]) OR hep b[tiab]) OR HBV[tiab])) AND ((((((mass screen*) OR test) OR tests) OR testing) OR case find*) OR screen*)

**Explanatory notes:** Search assembled using the ‘builder function’. MeSH terms avoided in an attempt to capture items not found through the MEDLINE search. When adding term ‘test*’, the search returns the message: “Wildcard search for 'test*' used only the first 600 variations. Lengthen the root word to search for all endings.” Thus, the truncated ‘test*’ search term further defined for the PubMed search.

**EMBASE Search Strategy**

1. Health Economics/

2. exp Economic Evaluation/

3. exp Health Care Cost/

4. pharmacoeconomics/

5. 1 or 2 or 3 or 4

6. (econom$ or cost or costs or costly or costing or price or prices or pricing or pharmacoeconomic$).ti,ab.

7. (expenditure$ not energy).ti,ab.

8. (value adj2 money).ti,ab.

9. budget$.ti,ab.

10. 6 or 7 or 8 or 9

11. 5 or 10

12. (metabolic adj cost).ti,ab.

13. ((energy or oxygen) adj cost).ti,ab.

14. ((energy or oxygen) adj expenditure).ti,ab.

15. 12 or 13 or 14

16. 11 not 15

17. animal/

18. exp animal experiment/

19. nonhuman/

20. (rat or rats or mouse or mice or hamster or hamsters or animal or animals or dog or dogs or cat or cats or bovine or sheep).ti,ab,sh.

21. 17 or 18 or 19 or 20

22. exp human/

23. human experiment/

24. 22 or 23

25. 21 not (21 and 24)

26. 16 not 25

27. exp virus hepatitis/

28. exp hepatitis b/

29. ((hepatitis or hep) adj b).ti,ab.

30. HBV.ti,ab.

31. 27 or 28 or 29 or 30

32. exp mass screening/

33. exp screening/

34. test$.mp.

35. case find$.mp.

36. screen$.mp.

37. 32 or 33 or 34 or 35 or 36

37. 26 and 31 and 37

**Explanatory notes:** Removed date and article type limits and terms relating to specific journals from NHS EED filter. ‘Hepatitis, viral, human/’ (MeSH term) is not an EMTREE term, thus ‘exp virus hepatitis’ used in place of this. The EMTREE term ‘exp screening/’ added to search strategy (note, this is not a MeSH term).

**CINAHL Plus Search Strategy**

S1        MH "Economics+"
S2        MH "Financial Management+"
S3        MH "Financial Support+"
S4        MH "Financing, Organized+"
S5        MH "Business+"
S6        S2 OR S3 or S4 OR S5
S7        S1 NOT S6
S8        MH "Health Resource Allocation"
S9        MH "Health Resource Utilization"
S10      S8 OR S9
S11      S7 OR S10
S12      TI (cost or costs or economic* or pharmacoeconomic* or price* or pricing*) OR AB (cost or costs or economic* or pharmacoeconomic* or price* or pricing*)
S13      S11 OR S12
S14      MH "Animal Studies"
S15  S13 NOT S14

S16 MH "Hepatitis, Viral, Human+”

S17 MH "Hepatitis B+"

S18 TI ((hepatitis or hep) N1 b) OR AB (hepatitis or hep) N1 b)

S19 TI (HBV) or AB (HBV)

S20 S16 OR S17 or S18 or S19

S21 MH "Health Screening+"

S22 TX mass screen*

S23 TX test*

S24 TX case find*

S25 TX screen*

S26 S21 OR S22 OR S23 OR S24 OR S25

S25 S15 AND S20 and S26

**Explanatory notes:** Removed date and article type limits from NHS EED filter.

**Cochrane library search strategy**

#1 MeSH descriptor: [Economics] explode all trees

#2 MeSH descriptor: [Costs and Cost Analysis] explode all trees

#3 MeSH descriptor: [Economics, Dental] explode all trees

#4 MeSH descriptor: [Economics, Hospital] explode all trees

#5 MeSH descriptor: [Economics, Medical] explode all trees

#6 MeSH descriptor: [Economics, Nursing] explode all trees

#7 MeSH descriptor: [Economics, Pharmaceutical] explode all trees

#8 (economic$ or cost or costs or costly or costing or price or prices or pricing or pharmacoeconomics$):ti,ab

#9 (expenditure$ not energy):ti,ab

#10 value for money:ti,ab

#11 budget$:ti,ab

#12 #1 or #2 or #3 or #4 or #5 or #6 or #7 or #8 or #9 or #10 or #11

#13 MeSH descriptor: [Hepatitis, Viral, Human] explode all trees

#14 MeSH descriptor: [Hepatitis B] explode all trees

#15 ((hepatitis or hep) adj b):ti,ab

#16 HBV:ti,ab

#17 #13 or #14 or #15 or #16

#18 MeSH descriptor: [Mass Screening] explode all trees

#19 test$

#20 case find$

#21 screen$

#22 #18 or #19 or #20 or #21

#23 #12 and #17 and #22

**Explanatory notes:** Adapted from MEDLINE search strategy.

**EconLit search strategy**

‘hepatitis’

**Explanatory notes:** As EconLit primarily indexes economics-related articles, terms used to filter economic evaluations in other databases are redundant. Search with single term, ‘hepatitis’ returned relatively few results and no additional relevant results were returned in Econlit when additional terms (e.g. HBV) were added; therefore the search strategy for these databases was left with this single term.

**Global Health search strategy**

1. exp economics/

2. "cost effectiveness analysis".sh.

3. "cost benefit analysis".sh.

4. "cost analysis".sh.

5. 1 or 2 or 3 or 4

6. exp hepatitis/

7. hepatitis B.sh.

8. ((hepatitis or hep) adj b).ti,ab.

9. HBV.ti,ab.

10. 6 or 7 or 8 or 9

11. exp screening/

12. test*.mp.

13. case find*.mp.

14. screen*.mp.

15. 11 or 12 or 13 or 14

16. 5 and 10 and 15

**Explanatory notes:** Adapted from MEDLINE search strategy.

**Open Grey search strategy**

(Hepatitis OR "hepatitis b" OR "hep b" OR "HBV") AND (screen* OR test* OR case find*)

**Explanatory notes:** Due to less-structured indexing in Open Grey, terms restricting the search to economic evaluations were omitted and only terms relating to hepatitis b or were screening included.

**CEA Registry search strategy**

‘Hepatitis b’

**Explanatory notes:** As the CEA registry primarily indexes economics-related articles, terms used to filter economic evaluations in other databases are redundant. Searching for hepatitis returned >100 results, premium (paid) access is required to access > 100 results, whereas searching for ‘hepatitis b’ returned <100 results, thus this term chosen instead.
