# Supplementary material for: A systematic review of hepatitis B screening economic evaluations in low- and middle-income countries
Source: BMC Public Health. 2018 Mar 20;18:373. doi: 10.1186/s12889-018-5261-8 (PMC5859762; doi:10.1186/s12889-018-5261-8)
Supplement: Supplementary file 2 — Rejected articles undergoing full-text review. (DOCX 27 kb) [file 12889_2018_5261_MOESM2_ESM.docx]

## Additional file 2. Rejected articles undergoing full-text review

**Under population criterion:**

1. Acar A, Kemahli S, Altunay H, Kosan E, Oncul O, Gorenek L, et al. The significance of repeat testing in Turkish blood donors screened with HBV, HCV and HIV immunoassays and the importance of S/CO ratios in the interpretation of HCV/HIV screening test results and as a determinant for further confirmatory testing. Transfus Med. 2010;20(3):152-9.
2. Allain JP, Opare-Sem O. Screening and diagnosis of HBV in low-income and middle-income countries. Nature Reviews Gastroenterology & Hepatology [Review]. 2016 Nov;13(11):643-653.
3. Bish DR, Bish EK, Xie SR, Slonim AD. Optimal selection of screening assays for infectious agents in donated blood. IIE Trans Healthc Sys Eng. 2011;1(2):67-90.
4. Chinkhumba J. Economics of blood screening: in search of an optimal blood screening strategy. Trop Doct. 2006;36(1):32-4.
5. Kania D, Sangare L, Sakande J, Koanda A, Nebie YK, Zerbo O, et al. A new strategy to improve the cost-effectiveness of human immunodeficiency virus, hepatitis B virus, hepatitis C virus, and syphilis testing of blood donations in sub-Saharan Africa: a pilot study in Burkina Faso. Transfusion. 2009;49(10):2237-40.
6. McNeil BJ, Dudley RA, Hoop B. A cost-effectiveness analysis of screening for hepatitis B surface antigen in India. Med Decis Making. 1981;1(4):345-59.

**Under intervention criterion:**

1. Management of liver cirrhosis. Lancet. 2014;383(9930):1694.
2. Aggarwal R, Ghoshal UC, Naik SR. Assessment of cost-effectiveness of universal hepatitis B immunization in a low-income country with intermediate endemicity using a Markov model. J Hepatol. 2003;38(2):215-22.
3. Ahmed A, Keeffe EB. Cost-effective evaluation of acute viral hepatitis. West J Med. 2000;172(1):29-32.
4. Aiello A, Guarino M, D'Ausilio A, Toumi M, Caporaso N, Morisco F. A real world cost analysis of serum HBsAg quantification test in patients with chronic hepatitis B: The case of the university of Naples federico II. Value in Health [Conference Abstract]. 2016 November;19 (7):A690.
5. Alavian SM. One swallow doesn't bring spring, reply to Khamesipour et al. Transfus and Apher Sci. 2012;46(1):103.
6. Atkins D, Ross D, Kelley M. Acting in the face of uncertainty. Ann Intern Med. 2014;161(4):300-1.
7. Brunette MF, Drake RE, Marsh BJ, Torrey WC, Rosenberg SD. Responding to blood-borne infections among persons with severe mental illness. Psych Serv. 2003;54(6):860-5.
8. Buffington J, Mast EE. Prevaccination screening for hepatitis B among sexually active adolescents and young adults. Clin Infect Dis. 1998;27(6):1562-3.
9. Diment J, Calmann M, Laperche S, Rouger P, Smilovici W, Herve P, et al. Alternatives to nucleic acid testing in the blood transfusion service. Lancet. 2002;360(9344):1518-20.
10. Dufour JF. Hepatology Highlights. Hepatology. 2014;59(5):1657-9.
11. Dziekan G, Chisholm D, Johns B, Rovira J, Hutin YJ. The cost-effectiveness of policies for the safe and appropriate use of injection in healthcare settings. Bull World Health Organ. 2003;81(4):277-.
12. Fairman KA, Curtiss FR. It's only a pharmacoeconomic model - Believe it or not. J Manag Care Pharm. 2008;14(1):83-5.
13. Grosse SD. Does newborn screening save money? The difference between cost-effective and cost-saving interventions. J Pediatr. 2005;146(2):168-70.
14. Kiberd B. Cost-effectiveness of routine nucleic acid testing in organ donors. Am J Transplant. 2014;14(4):979-80.
15. Nayagam S, Conteh L, Sicuri E, Shimakawa Y, Lemoine M, Hallett TB, et al. Community-based screening and treatment for chronic hepatitis B in sub-Saharan Africa - Authors' reply. The Lancet Global Health [Letter]. 2017 01 Jan;5(1):e35.
16. Qin G, Shao JG. Community-based screening and treatment for chronic hepatitis B in sub-Saharan Africa. The Lancet Global Health [Letter]. 2017 01 Jan;5(1):e34.
17. Sum SS, Wong DK, Yuen JC, Lai CL, Yuen MF. Comparison of the COBAS TaqMan HBV test with the COBAS Amplicor monitor test for measurement of hepatitis B virus DNA in serum. J Med Virol. 2005;77(4):486-90.

**Under comparator criterion:**

1. Allain JP. Screen-and-treat for chronic hepatitis B: an overdue issue for sub-Saharan Africa. The Lancet Global Health [Note]. 2016 01 Aug;4(8):e507-e508. Fisher SA, Hennink M. Screening before immunisation against hepatitis B--is it cost-effective? S Afr Med J. 1989;76(7):387.
2. Das HS, Patra UC, Jena SK, Behera S, Sahoo RP, Mallick A. Free screening and vaccination for hepatitis B in health care workers of S C B Medical College, Cuttack, Odisha. Indian Journal of Gastroenterology [Conference Abstract]. 2016 November;35 (1 Supplement):A57-A58.
3. Fisher SA, Hennink M. Screening before immunisation against hepatitis B--is it cost-effective? S Afr Med J. 1989;76(7):387.
4. Griffiths I, Dornan J, Aston I. Current practice in testing for hepatitis B virus carriage in non-seroconverters. Occ. Med. 2001;51(8):517-8.
5. Isa MA, Bello HS, Shettima A, Allamin IA. Prevalence of serological marker for chronic hepatitis B virus among patients attending Sokoto specialist hospital, Sokoto, Nigeria. J Microbiol. Biotech. Res. 2013;3(3):122-5.
6. Karim Rumi MA, Begum K, Sawkat Hassan M, Munir Hasan SM, Golam Azam M, Nadim Hasan K, et al. Detection of hepatitis B surface antigen in pregnant women attending a public hospital for delivery: Implication for vaccination strategy in Bangladesh. Am J Trop Med Hyg. 1998;59(2):318-22.
7. Khan NR, Sadiq F. Prenatal screening for hepatitis B virus. Int J Gynaecol Obstet. 1996;55(1):79-80.
8. Mathur M, Wanjari K, Turbadkar D. Seroprevalence of HIV, hepatitis C and hepatitis B in multitransfused thalassemics. Ind J Med Microbiol. 2008;26(2):205-6.
9. Muselmani W, Habbal W, Monem F. Significance of screening antibodies to hepatitis B virus core antigen among Syrian blood donors. Transfus Med. 2013;23(4):265-8.
10. Owusu-Ofori S, Temple J, Sarkodie F, Anokwa M, Candotti D, Allain J. Predonation screening of blood donors with rapid tests: implementation and efficacy of a novel approach to blood safety in resource-poor settings. Transfusion. 2005;45(2):133-40.
11. Portas L, Langham A, Taylor R. MSM screening in saunas-is it worth it? Sexually Transmitted Infections [Conference Abstract]. 2016;92:A33.
12. Weber P, Eberle J, Bogner J, Schrimpf F, Jansson V, Huber-Wagner S. Is there a benefit to a routine preoperative screening of infectivity for HIV, hepatitis B and C virus before elective orthopaedic operations? Infection. 2013;41(2):479-83.

**Under outcome criterion:**

1. Allain JP, Owusu-Ofori S. Occult hepatitis B infection in blood donors. Vox Sang. 2008;94(2):162-4.
2. Alswaidi FM, O'Brien SJ. Premarital screening programmes for haemoglobinopathies, HIV and hepatitis viruses: review and factors affecting their success. J Med Screen. 2009;16(1):22-8.
3. Andre F. Hepatitis B: a comprehensive prevention, diagnosis, and treatment program - past, present, and future. (Hepatitis disease management: Integrating prevention, diagnosis, and therapeutics). J Gastroenterol Hepatol. 2004;19:S1-S4.
4. Chen DS. Public health measures to control hepatitis B virus infection in the developing countries of the Asia-Pacific region. J Gastroenterol Hepatol. 2000;15 :E7-10.
5. Chen DS, Locarnini S, Wait S, Bae SH, Chen PJ, Fung JY, et al. Report from a Viral Hepatitis Policy Forum on implementing the WHO Framework for Global Action on viral hepatitis in North Asia. J Hepatol. 2013;59(5):1073-80.
6. Dankner WM, Dixon SD, Lane TA, Moore T, Silverman NS, Wapner RJ. Hepatitis B in a prenatal population. JAMA. 1993;269(5):589-90.
7. Li L, Chen P, Chen M, Chak K, Lin K, Tsai SL. A pilot study for screening blood donors in Taiwan by nucleic acid amplification technology: detecting occult hepatitis B virus infections and closing the serologic window period for hepatitis C virus. Transfusion. 2008;48(6):1198-206.
8. Mehr MT, Uddin S, Ul Iman N. Screening of HBV, HCV and HIV before endoscopy. JMedSci. 2015;23(2):104-8.
9. Muller J, Hainsworth M, Terry T. Screening before immunisation against hepatitis B--is it cost-effective? S Afr Med J. 1989;75(10):504-5.
10. Nayagam S, Thursz M, Sicuri E, Conteh L, Wiktor S, Low-Beer D, et al. Requirements for global elimination of hepatitis B: a modelling study. The Lancet Infectious Diseases. 2016 Dec;16(12):1399-1408.
11. Sahni M, Jindal K, Abraham N, Aruldas K, Puliyel JM. Hepatitis B immunization: cost calculation in a community-based study in India. Indian J Gastroenterol. 2004;23(1):16-8.
12. Sriprakash I, Anil TP. Routine prenatal screening of Indian women for HBsAg: benefits derived versus cost. Trop Doct. 1997;27(3):176-7.
13. van Hulst M, Hubben GA, Sagoe KW, Promwong C, Permpikul P, Fongsatitkul L, et al. Web interface-supported transmission risk assessment and cost-effectiveness analysis of postdonation screening: a global model applied to Ghana, Thailand, and the Netherlands. Transfusion. 2009;49(12):2729-42.

**Under setting criterion:**

1. Inactivated hepatitis B virus vaccine. Recommendation of the immunization practices advisory committee. Ann Int Med. 1982;97(3):379-83.
2. ICPs refine blanket testing for patients, health care workers. Hosp Infect Control. 1993;20(9):125-8.
3. Alderman EM, Shapiro A, Spigland I, Bashir M, Fox AS. Is prevaccination screening for hepatitis B among sexually active adolescents cost-effective? Clin Infect Dis. 1998;26(6):1459-60.
4. Armbruster B, Brandeau ML. Cost-effective control of chronic viral diseases: finding the optimal level of screening and contact tracing. Math Biosci. 2010;224(1):35-42.
5. Best L. Screening for hepatitis B in Pregnancy. Southampton; Wessex Institute for Health Research and Development:1996.
6. Bish EK, Ragavan PK, Bish DR, Slonim AD, Stramer SL. A probabilistic method for the estimation of residual risk in donated blood. Biostatistics. 2014;15(4):620-35.
7. Blostein J, Clark PA. Cost-effectiveness of preimmunization hepatitis B screening in high-risk adolescents. Public Health Rep. 2001;116(2):165-8.
8. Busch MP. Should HBV DNA NAT replace HBsAg and/or anti-HBc screening of blood donors? Transfus Clin Biol. 2004;11(1):26-32.
9. Buti M, Crespo J, Esteban R, Torres C, Oyaguez I, Casado MA. Cost-effectiveness analysis of hepatitis B virus screening to prevent hepatitis B virus reactivation in patients receiving rituximab based chemotherapy for hematologic malignancy. Journal of Hepatology [Conference Abstract]. 2016 April;1):S596.
10. Cairns JA, Shackley P. Assessing value for money in medical screening. J Med Screen. 1994;1(1):39-44.
11. Christian SS, Duff P. Is universal screening for hepatitis B infection warranted in all prenatal populations? Obstet & Gynecol. 1989;74(2):259-61.
12. Christopher PJ. Should all pregnant women be screened for hepatitis B surface antigen? Med J Aust. 1989;150(6):346, 8.
13. Dienstag JL, Silverstein MD, Mulley AG. The cost-effectiveness of hepatitis B vaccine. J Infect. 1983;7 Suppl 1:81-4.
14. Diez Redondo Ma P, Almaraz A, Jimenez Rodriguez-Vila M, Santamaria A, De Castro J, Torrego JC, et al. Comparison of two vaccination strategies against hepatitis A and B in patients with chronic hepatitis C. [Spanish, English]. Rev Esp Enferm Dig. 2009;101(4):265-74.
15. Hankins DG, Ebert KD, Siebold CM, Fuller TK, Frascone RJ, Campion BC. Hepatitis B vaccine and hepatitis B markers: cost effectiveness of screening prehospital personnel. Am J Emerg Med. 1987;5(3):205-6.
16. Hashimoto F. Cost effectiveness of serotesting before hepatitis vaccination. N Engl J Med. 1982;307(8):503.
17. Hay AE, Meyer RM. Hepatitis B, rituximab, screening, and prophylaxis: effectiveness and cost effectiveness. J Clin Onc. 2012;30(26):3155-7.
18. Heijtink RA, Kruining J, Schalm SW, Masurel N. Hepatitis B: prevaccination screening of individuals simultaneously positive for HBsAg and anti-HBs in one assay. Lancet. 1983;1(8332):1050-1.
19. Hoerger TJ, Bradley C, Schillie SF, Reilly M, Murphy TV. Cost-Effectiveness of Ensuring Hepatitis B Protection for Previously Vaccinated Healthcare Personnel. Infection Control Hosp Epidemiol. 2014;35(7):845-54.
20. Hopkins R, Ross S, Jordan T, Watt AD. Improved economics of HBsAg screening with commercial radioimmunoassay reagents. J Clin Pathol. 1980;33(1):19-23.
21. Jacobson JJ, La Turno DE, Johnston FK, Shipman C, Jr. Cost effectiveness of prevaccination screening for hepatitis B antibody. J Dent Educ. 1987;51(2):94-7.
22. Little RF, Brenner ER, Macera CA, Jackson KL. Cost-effective pre-vaccine screening of hepatitis B infection in hospital workers: a seroepidemiological study. J S C Med Assoc. 1988;84(8):409-13.
23. Lo Re V, 3rd. Economic analysis of hepatitis B screening and treatment. Clin Infect Dis. 2011;52(11):1307-9.
24. Malek M, Davey P. Economics of mandatory HIV and hepatitis B virus testing for healthcare workers performing surgical procedures. Pharmacoeconomics. 1993;4(6):401-4.
25. Pereira A. Health and economic impact of posttransfusion hepatitis B and cost-effectiveness analysis of expanded HBV testing protocols of blood donors: a study focused on the European Union. Transfusion. 2003;43(2):192-201.
26. Perrillo RP. Screening of health care workers before hepatitis B vaccination: more questions than answers. Ann Int Med. 1985;103(5):793-5.
27. Ringwald J, Mertz I, Zimmermann R, Weisbach V, Strasser E, Achenbach S, et al. Hepatitis B virus vaccination of blood donors--what costs may be expected? Transfus Med. 2005;15(2):83-92.
28. Schupp Christian S, Duff P. Is universal screening for hepatitis B infection warranted in all prenatal populations? Obstet Gynecol. 1989;74(2):259-61.
29. Van der Have M, Oldenburg B, Fidder H, Belderbos TD, Van Der Scheer F, Siersema PD, et al. Extensive screening for opportunistic infections prior to biological therapy in patients with crohn's disease is not cost-effective. Gastroenterology. 2012;1):S734.
30. Van der Have M, Oldenburg B, Fidder HH, Belderbos TDG, Siersema PD, Van Oijen MGH. Optimizing screening for tuberculosis and hepatitis B prior to starting tumor necrosis factor- alpha inhibitors in Crohn's disease. Dig Dis Sci. 2014;59(3):554-60.
31. Wong WW, Hicks LK, Tu HA, Pritchard KI, Krahn MD, Feld JJ, et al. Hepatitis B virus screening before adjuvant chemotherapy in patients with early-stage breast cancer: a cost-effectiveness analysis. Breast Cancer Res Treat. 2015;151(3):639-52.

**Under ‘not full economic evaluation’ criterion**

1. Allain JP, Opare-Sem O, Sarkodie F, Rahman R, Owusu-Ofori S. Deferred donor care in a regional hospital blood center in Ghana. Transfusion. 2009;49(4):669-75.
2. Anderson S, Harper L, Dionne-Odom J, Ekane GH, Tita A. Prevention of hepatitis B in sub-Saharan Africa: A decision analytic model for the birth dose vaccine. American Journal of Obstetrics and Gynecology [Conference Abstract]. 2017 January;216 (1 Supplement 1):S234.
3. Botha JF. SAGE guidelines committee. S Afr Gastroenterol Rev. 2007;5(3):30-1.
4. Boyles S. Is universal better than selective immunization in developing world? Vaccines (HBV). Hepatitis Wkly. 1998:7-8.
5. Chongsuvivatwong V. A simplified financial cost-effectiveness analysis of programs for prevention of hepatitis B accidental inoculation among hospital personnel in Thailand. Southeast Asian J Trop Med Public Health. 1989;20(2):189-93.
6. Custodio H, Mirza A, Salahuddin A, Ferguson K, Rathore M. Cost-effectiveness of hepatitis B serology testing before hepatitis B vaccination in the Akha tribal children in northeastern Thailand. Pediatr Infect Dis J. 2011;30(8):725.
7. Hamid S, Iqbal A. Hepatitis B immunization of hospital employees in an endemic area: should we screen? Infect Control Hosp Epidemiol. 1997;18(10):680-1.
8. Hillman AL, et al. Cost Effectiveness of Hepatitis B Immunisation Strategies. Pharmacoeconomics. 1994;5(2):85-7.
9. Iber FL. Prevention of posttransfusion hepatitis: cost-effective or not? JAMA. 1981;246(20):2356.
10. Kane MA, Hadler SC, Margolis HS, Maynard JE. Routine prenatal screening for hepatitis B surface antigen. JAMA. 1988;259(3):408-9.
11. Khurana V, Kar P, Mansharamani N, Jain V, Kanodia A. Differences in hepatitis B markers between clinical and preclinical health care personnel. Trop Gastroenterol. 1997;18(2):69-71.
12. Koplan JP. Prenatal screening for hepatitis B. JAMA. 1988;259(23):3408-9.
13. Koretz RL. Universal prenatal hepatitis B testing: is it cost-effective? Obstet Gynecol. 1989;74(5):808-14.
14. Ozkan S, Atak A, Bozdayi G, Turkcuoglu S, Maral I. Community-based research: cost of the tests used for anti-HBc total seropositivity only and hepatitis B screening. Trans R Soc Trop Med Hyg. 2010;104(12):782-6.
15. Schoub BD, Johnson S, McAnerney J, Blackburn NK, Padayachee GN. Exposure to hepatitis B virus among South African health care workers--implications for pre-immunisation screening. S Afr Med J. 1991;79(1):27-9.
16. Shivaram C. Half a decade of mini-pool nucleic acid testing: cost-effective way for improving blood safety in India. Asian J. Transfus. Sci. 2014;8(1):35-8.
17. Thursz MR. Screening and treatment of hepatitis B virus to prevent liver cancer in Africa. Hepat Oncol. 2015;2(2):105-9.

**No full-text article available in English**

1. Opare-Sem O, Owusu-Ofori S, Allain JP. A novel approach to blood safety: pre-donation screening of blood donors for viral markers. Africa Sanguine. 2002;5(2):12-6.

*(Could not locate full-text in spite of attempting through three university online libraries – the School, Curtin University and the University of Tasmania – , via the InterLoans service at the School, contacting the journal editors and the corresponding author (email accessed via similar study by Owusu-Ofori et al., ref 27 above).*

1. Vernier, M. Screening for hepatitis A, B, C and E among expatriate business travelers [Thesis]. Marseille; Marseille Université: 2012.
