# Supplementary material for: A systematic review of hepatitis B screening economic evaluations in low- and middle-income countries
Source: BMC Public Health. 2018 Mar 20;18:373. doi: 10.1186/s12889-018-5261-8 (PMC5859762; doi:10.1186/s12889-018-5261-8)
Supplement: Supplementary file 3 — Data extracted from included studies. (DOCX 71 kb) [file 12889_2018_5261_MOESM3_ESM.docx]

## Additional file 3. Data extracted from included studies (in alphabetical order, by first author surname).

| **Title & Abstract** | **Introduction** | **Methods** | **Results** | **Discussion** | **Other** |
| --- | --- | --- | --- | --- | --- |
| Abidi *et al.* (2004) (30)  **Identification in title as an economic evaluation?**  Yes, “an economic analysis”.  **Abstract with clear sections for objectives, perspective, setting, methods, results and conclusions?**  Yes | **Statement addressing context for the study?**  No single statement, but background section introduces context.  **Study question:**  No specific question, rather aim to perform “an economic analysis of premarriage prevention of hepatitis B transmission”.  **Relevance to health policy:**  Aimed at health policy makers “in Iran and those countries with similar demographic conditions”. | **Population & subgroups:** Premarriage couples (in context of assumed no premarital sex).  **Setting:** Iran.  **Perspective:**  Health care system in base case. ‘Societal perspective’ for sensitivity analysis assessing impact of chronic liver disease (CLD).  **Interventions compared:**   1. Screening all pre-marriage couples for HBsAg, providing prevention protocol for serodiscordant couples (HBV vaccination, HBIG injection, condoms, HB surface antibody titre after 3^rd^ HBV vaccine and 4^th^ vaccine if too low). 2. As for 1) but with screening of HB core antibody for HBsAg –ve partner and prevention protocol only if –ve for both markers 3. No screening, no prevention.   **Time Horizon:** Not stated for base case. 35 years for sensitivity analysis (assumed marriage at age 25, costs of CLD for 10 years from age 50).  **Discount rate:** 3% for costs for sensitivity analysis considering effect of CLD.  **Outcome measures:** Chronic infections averted for base case. Cost of CLD to determine threshold below which screening measures were not ‘cost saving’.  **Modeling or study-based design:** Decision tree modeling. Transition probabilities: searching Iran “accurate” medical literature, via PubMed and expert opinion (5 gastroenterologists), if literature value unavailable.  **Preference measures for outcomes:** Not considered.  **Source(s) for resource use:** Direct costs – government and health facility sources. Indirect costs – assumed to be zero.  **Date(s) of resource use, currency, year:** Cost data sourced from year study undertaken (2003). Costs expressed in USD and Iranian Rials (1$US = 8,300 Rials, conversion mid 2003).  **Justification for type of decision-analytic model used?** In discussion, lack of data for costs of CLD used to explain not using a Markov Model or clear willingness-to-pay for screening. Figure provided.  **Methods for analysis described?**  Threshold analysis to consider cost of CLD below which screening interventions would be ‘cost saving’. One-way sensitivity analyses with transition probabilities, two-way sensitivity analysis with linked prevalence of HBsAg and of HB core antibody in general population. | **Values, ranges, references used reported?**  Transition probabilities: values, ranges and references reported.  Cost data: values, no range, sourced from unpublished data, thus unreferenced.  **Results of main analysis:**   1. $US 202/ chronic infection averted. 2. $US 197/chronic infection averted.   **Sensitivity analysis/es results:**  One-way sensitivity:   1. Range: $US67 - $US330. 2. Range: $US66 - $309.   Two-way sensitivity: “strategy two always cheaper”.  ‘Worst case scenario’ (minimum effectiveness, maximum costs over range):   1. $US 296 2. $US 293   Threshold analysis (discounted, cost of CLD above which screening strategies are cost saving). :   1. $US 2,818 2. $US 2,747 | **Key findings:**  Though data lacking, United States cost data on CLD would suggest strategy 1 and 2 are “cost saving”, in terms of cost avoided by preventing CLD.  S2 always “cheaper” than S1 in this analysis.  **Study strengths (author reported):**  Some consideration of the affect of compliance to the prevention protocol via sensitivity analysis.  **Study limitations (author reported):**  Limited generalisability to contexts where pre-marital sex is more common. Data lacking for costs of acute and CLD. Acute infection not considered, only chronic infection. No consideration of vertical transmission (to infants born to couples).  **Study conclusions:**  Prevention strategy “seems cost saving”.  Further research should assess costs associated with HBV infection, particularly via CLD. | **Funding:**  Research Center for Gastroenterology & Liver Disease, Shaheed Beheshti University of Medical Sciences, Tehran, Iran.  **Conflicts of interest:**  None declared. |

| **Title & Abstract** | **Introduction** | **Methods** | **Results** | **Discussion** | **Other** |
| --- | --- | --- | --- | --- | --- |
| Aggarwal and Naik (1994)* (31)  **Identification in title as an economic evaluation?**  No.  **Abstract with clear sections for objectives, perspective, setting, methods, results and conclusions?**  Abstract not separated into clear sections. No clear objective or perspective, setting is identified, methods are not specified, results (though no exact figures) and conclusions are given. | **Statement addressing context for the study?**  **“**We examined the problem of HBV infection is India with a view to recommending the best course for decreasing its prevalence”.  **Study question:**  [relevant to this review]: “What would be the most cost-effective strategy – selective or universal immunisation?”.  **Relevance to health policy:**  No clear target audience identified. | **Population & subgroups:** Pregnant women.  **Setting:** India.  **Perspective:** No explicit statement.  **Interventions compared:**   1. Universal immunisation with no screening. 2. Screening pregnant women for HBsAg, immunising infants born to HBsAg +ve mothers.   **Time Horizon:** Not stated.  **Discount rate:** No discounting.  **Outcome measures:** Number of carriers prevented/10,000 infants born.  **Modeling or study-based design:**  Simple modeling study, with data sourced from multiple sources as part of the authors’ review.  **Preference measures for outcomes:** Not used.  **Source(s) for resource use:** An ‘international estimate’ for vaccine procurement and administration costs. Costs per infant vaccinated estimated as double for strategy 2 as “…with this policy, economies of scale cannot be achieved”.  **Date(s) of resource use, currency, year:** Some cost data sourced from paper published in 1993, prices in $US, not stated what year.  **Justification for type of decision-analytic model used?** Rationale for costs and projected numbers of carriers prevented provided, no specific justification for type of modelling used. Model figure provided.  **Methods for analysis described?**  No sensitivity analyses performed. | **Values, ranges, references used reported?**  Values reported in tables, no ranges for either cost of projected carrier numbers. Sources of most values provided in the text. No references for costs associated with screening tests (“assumed as total of $US2 per pregnant mother). No references for expected ‘protective efficacy’ of universal (92%) or selective (12%) vaccination policies.  **Results of main analysis:**   1. Universal immunisation: 341.2 carriers/10,000 infants born prevented, total cost $US 43,000, therefore $US126/carrier prevented. 2. Screening followed by selective immunisation: 44.8 carriers/10,000 infants born prevented, total cost $US 22,182, therefore $US 495/carrier prevented.   **Sensitivity analysis/es results:**  Not applicable. | **Key findings:**  Universal immunisation is more costly overall, but less costly per carrier prevented, when compared to selective immunisation of infants.  **Study strengths (author reported):**  None stated.  **Study limitations (author reported):**  None stated.  **Study conclusions:**  “We hope that this article will create an awareness about the need for an HBV vaccination strategy, will initiate debate in this important are and, finally lead to evolution of an effective strategy to combat this serious infection in our population”. | **Funding:**  None mentioned.  **Conflicts of interest:**  None mentioned. |

* This is a review article comprising a cost-effectiveness analysis, thus the usual structure of an economic evaluation (e.g. as per the CHEERS statement) is not followed; data is extracted from the paper from relevant sections, not necessarily corresponding to those on the data extraction form. Cost data were assumed to be in the year of publication (1994).

| **Title & Abstract** | **Introduction** | **Methods** | **Results** | **Discussion** | **Other** |
| --- | --- | --- | --- | --- | --- |
| Chen *et al.* (2016) (32)  **Identification in title as an economic evaluation?**  Yes, “Economic evaluation…”  **Abstract with clear sections for objectives, perspective, setting, methods, results and conclusions?**  No abstract provided. | **Statement addressing context for the study?**  “Most of the previous studies did not include screening for pregnant women and HBIG injection.”.  **“**The objective  of this study is to provide an updated and comprehensive  economic evaluation of infant HepB vaccination with 10ug vaccine and HBIG.”  **Study question:**  No research question, objective provided above.  **Relevance to health policy:**  “Results from this study will be  important and useful for making future policy decisions  regarding HepB vaccination.” | **Population & subgroups:** the 2013 birth cohort of 16, 437, 439, divided into 3 subgroups by mothers’ HBV infection status:   - Both HBsAg +ve and HBeAg +ve - HBsAg +ve and HBeAg –ve - HBsAg –ve   These were selected as the interventions were aimed at newborns following screening of pregnant women.  **Setting:** China.  **Perspective:** No explicit statement. Assesses costs from direct and societal perspective.  **Interventions compared:**   1. Without immunisation program 2. Hepatitis B immunisation program 3. Hepatitis B immunisation program combined with HBIG for infants of HBsAg +ve mothers   (3) compared to (1) and (3) compared to (2)  **Time Horizon:** Lifetime of infants in model.  **Discount rate:** 3%, not justified but consistent with Gates Reference Case.  **Outcome measures:**   - Number of HBV infections prevented - Number of early deaths from HBV prevented - Related direct and societal program costs of averted illness - Direct and societal net present value (NPV) - Direct and societal benefit cost ratios (BCR)   **Modeling or study-based design:**   - Decision tree - Markov model   **Preference measures for outcomes:** Not applicable.  **Source(s) for resource use:**   - Published literature - Surveillance data - Sero-survey data - Study data - Expert consensus - Large computerized data sets - Prevention of Mother-to-child transmission of HBV report, China. - Chinese center for Disease control and prevention   **Date(s) of resource use, currency, year:**  Costs are adjusted for 2013 US dollars using general Consumer Price Indices (CPI) and medical CPI.    **Justification for type of decision-analytic model used?** No justification; model figure is provided.  **Methods for analysis described?**   - The decision tree modelling - Compared disease and economic outcomes over the lifetime of a birth cohort between HepB vaccination combined with HBIG and No HepB vaccination - Evaluated the impact of HBIG - Direct medical costs, direct non-medical costs, societal costs and costs associated with the infant HepB immunization program were calculated - Benefits of immunization were quantified as the savings in direct and societal costs that accrue from averting morbidity and mortality by vaccination - NPV is the discounted benefit from the immunization program minus the discounted immunization program cost - BCR is equal to the discounted benefit divided by the discounted immunization program cost - The immunization program to be cost saving if BCR≥1.0 - One way sensitivity analyses were performed; reported for (3) versus (1) comparison. | **Values, ranges, references used reported?**  These are provided in Table 4 of the report. As probabilistic sensitivity analysis was not undertaken, distributions were not provided.  **Results of main analysis:**  If no universal infant HepB vaccination:   - About 12.89 million HBV new infection - 599,518 early deaths from HBV - A direct cost of $12.09 billion - A societal cost of $46.93 billion   Compared with no HepB vaccination (1), HepB vaccination +HBIG (3) could prevent:   - 96.91% (12.49 million) of HBV infections - 96.11% (0.58 million) of early deaths from HBV related diseases - The related direct and societal cost of averted illness would be $11.68 billion and $45.14 billion, respectively - The direct and societal net present value (NPV) of the program would be $11.5 billion and $44.9 billion, respectively - The direct and societal benefit cost ratios (BCRs ) would be 61.3 and 193.2, respectively - Compared with HepB vaccination (2), HepB vaccination+HBIG (3) could prevent: - 3,500 HBV infections - The direct and societal BCRs for the HBIG administration were 0.4 and 2.7, respectively   **Sensitivity analysis/es results:**   - BCRs remaining above 1.0 regardless of changes in key parameter values for (3) versu s (1) comparison. - Most influence parameters: the costs of health outcomes, discount rate, and administration costs - The worst-case scenario, the model using lower bound estimates of disease costs obtained from a western city, and the direct and societal BCRs were 30.8 and 161.5, respectively, much lower than the base case BCRs - Direct and societal BCRs would decrease to 44.3 and 147.4, respectively, if the administration costs were doubled. - No reported sensitivity analysis for (3) versus (2) comparison. | **Key findings:**  Universal infant HepB vaccination combined with one dose of HBIG would:   - Prevent more than 12 million HBV infections, and 576,209 cases of HBV related deaths compared with no HepB vaccination (1). - Save direct and societal cost of $11.68 billion and $45.14 billion, respectively and have high BCRs (61.3 and 193.2, respectively).   Sensitivity analyses indicated our model is very stable with BCRs remaining above 1.0 regardless of changes in parameter values for (3) versus (1) comparison.  **Study strengths (author reported):**   - Study obtained important parameter values from national surveillance system which more reliable - The HBV epidemic parameters in pregnant women was obtained from the most current national sero survey data.   **Study limitations (author reported):**   - Study did not include additional work time loss costs which might be an understatement of the costs of illness (e.g. through informal/family care). - China has substantial geographic differences across regions, it was difficult to assume that some parameters were nationally representative - Study did not include effectiveness of antiviral drugs - In Markov model, it was assumed that the transition probabilities from one health state to another were not age-dependent.   **Study conclusions:**  The current infant HepB **“**In summary, the current infant HepB immunization program  in China was effective in preventing new HBV infection,  and it could reduce HBV related morbidity and mortality while  also reducing health care costs. These results supported China’ s  health policy makers in continuing central and regional funding  for vaccination of high risk newborns combined with  HBIG.” | **Funding:**  The Chinese Ministry of Science and Technology Program for Important Infectious Diseases Control and Prevention  **Conflicts of interest:**  No potential conflicts of interest were disclosed. |

| **Title & Abstract** | **Introduction** | **Methods** | **Results** | **Discussion** | **Other** |
| --- | --- | --- | --- | --- | --- |
| Guidozzi *et al.* (1993) (33)  **Identification in title as an economic evaluation?**  No.  **Abstract with clear sections for objectives, perspective, setting, methods, results and conclusions?**  Abstract not separated into clear sections. No clear statement of objective, perspective, results of cost-effectiveness part of study. Other prevalence) results and conclusions are provided and the setting is introduced. | **Statement addressing context for the study?**  “In South Africa…the financial restraints have precluded routine screening for HBV in pregnant mothers”.  **Study question:**  No question, objective of study is mainly to determine HBV prevalence via screening.  **Relevance to health policy:**  “…to determine the cost-effectiveness of prenatal screening and immunisation for HB by routinely screening all antenatal patients…”. No clear target audience for analysis. | **Population & subgroups:** Pregnant women (White, Black and Asian).  **Setting:** Johannesburg Hospital, South Africa.  **Perspective:** No explicit statement.  **Interventions compared:**  From 1 Jan to 1 November 1990: Screening for HBsAg, hepatitis B surface antibody, HBeAg, H B e antibody, hepatitis B core antibody.  Infants born to HBsAg +ve mothers were given HB prophylaxis (not specified what this consisted of).  From 1 Jan to 31 August 1991: Screening for HBsAg only, further screening for other markers described above if +ve.  Infants born to HBsAg +ve mothers were vaccinated.  **Compared to “**worst-case scenario” of no screening.  **Time Horizon:** Not specified.  **Discount rate:** No discounting.  **Outcome measures:** Infections averted.  **Modeling or study-based design:**  Authors estimate cases prevented, assuming mothers who are HBsAg and HBeAg +ve, or HBsAg +ve and HBe antibody -ve will transmit infection to their infants in 90-100% and 10-12% of case respectively, references provided.  **Preference measures for outcomes:** Not applicable.  **Source(s) for resource use:** Source of costs not mentioned.  **Date(s) of resource use, currency, year:** South African Rand (R), date not reported.  **Justification for type of decision-analytic model used?** No justification, ‘creation of a worst-case scenario’.  **Methods for analysis described?**  Only for screening part of study, no description of economic analysis in the methods section, results of economic evaluation only reported in the discussion of the article rather than the main results section. | **Values, ranges, references used reported?**  Reported in discussion section of article, no range provided, references given for probability of infection, reference to other prevalence studies in South Africa, no references for cost data.  **Results of main analysis:**  Estimation of 7 HBV infections averted (1/500 births; 3,469 women screened), total cost of R 86,690, costs per case averted (compared to no screening) of R 12,384 with cost of each HBsAg screening test estimated as R 24.99.  **Sensitivity analysis/es results:** None mentioned. | **Key findings:**  Screening compared to no screening costs R 12,384/HBV case averted, based on hypothetical scenario (model).  This finding does not agree with epidemiological data from South Africa (from this study or from another study cited in the paper ) – suggesting that vertical transmission is less common in South Africa than in other settings.  **Study strengths (author reported):**  None reported.  **Study limitations (author reported):**  None reported.  **Study conclusions:**  Appears the routine screening in a South African urban hospital in impractical and not cost-effective, routine universal immunisation is more appropriate and will also reduce horizontal transmission. | **Funding:**  Not reported  **Conflicts of interest:**  Not reported |

| **Title & Abstract** | **Introduction** | **Methods*** | **Results*** | **Discussion** | **Other** |
| --- | --- | --- | --- | --- | --- |
| Lansang *et al.* (1989) (34)  **Identification in title as an economic evaluation?**  Yes, “cost-effectiveness analysis”.  **Abstract with clear sections for objectives, perspective, setting, methods, results and conclusions?**  Abstract not separated into separate sections. Objective, descriptive results and conclusions specified; perspective and setting not specified. | **Statement addressing context for the study?**  No explicit statement, context for study introduced in background section.  **Study question:**  No study question.  **Relevance to health policy:**  “This paper evaluates the cost-effectiveness of using this rapid epidemiologic tool in HBV control programmes, in contrast to screening programmes using conventional methods or strategies advocating mass immunization”.  No clear target audience specified. | **Population & subgroups:** Pregnant women.  **Setting:** Philippines.  **Perspective:** No explicit statement.  **Interventions compared:**   1. Screening with ‘finger prick’ sample, administering HBV vaccine to infants of women with HBsAg titres > 2^2.5^. 2. Screening with venous blood sample tested via reverse passive haemagglutination and administering HBV vaccine to infants of women with HBsAg titres> 2^11.5^. 3. Screening with venous blood sample tested for both HBsAg and HBeAg by radioimmunoassay, administer HBV vaccine to neonates of women who are HBeAg +ve. 4. Mass immunisation of all infants at birth with no screening.   Also considered strategies 1 to 3 with and without administering HBIG in addition to HBV vaccine.  **Time Horizon:** Not specified.  **Discount rate:** No discounting.  **Outcome measures:** Outcome of ‘utility’, defined as 1 minus the seroconversion rate from giving HBV vaccine +/- HBIG, states that these are ‘known’ but does not mention source. Utility for ‘no vaccine’ derived from “…various vaccine trials…” (unreferenced).  **Modeling or study-based design:**  Decision tree modeling (hypothetical cohort of 1 pregnant woman).  **Preference measures for outcomes:** Not used (in spite of the term ‘utility’, see above).  **Source(s) for resource use:** Costs were “estimated”; sources are not mentioned. HBV vaccine costed at 25% of then commercial cost due to expectation that this would decrease (Thailand and Indonesia cited as examples supporting this assumption). Direct costs only considered, cost of staff time (“manpower”) included.  **Date(s) of resource use, currency, year:** Philippine Pesos (20 peso = 1 $US). Date of resource estimation or currency conversion not specified.  **Justification for type of decision-analytic model used?** No specific justification for model choice. Figure provided.  **Methods for analysis described?**  See results section. | **Values, ranges, references used reported?**  Values reported in table form and rationale provided for calculation of costs and utilities as an appendix. References or ranges not provided.  **Results of main analysis:**  Cost-effectiveness defined as expected cost (EC in pesos)/expected ‘utility’ (EU) per person)   1. a) HBV vaccine alone: EC = 12, EU = 0.9, EC/EU = 13.33   b) HBV vaccine & HBIG: EC = 33, EU = 0.9, EC/EU = 36.67   1. a) HBV vaccine alone: EC = 12, EU = 0.89, EC/EU = 14.04   b) HBV vaccine & HBIG: EC = 23, EU = 0.89, EC/EU = 25.84   1. a) HBV vaccine alone: EC = 93, EU = 0.90, EC/EU = 103.33   b) HBV vaccine & HBIG: EC = 100, EU = 0.9, EC/EU = 111.1  4) EC = 150, EU = 0.95, EC/EU = 157.89.  **Sensitivity analysis/es results:**  Threshold analysis showed that if the HBV vaccine cost dropped below 87 pesos (from 150) strategy 3 would become the least cost-effective. Adjusting the efficacy of HBV vaccination in infants born to HBeAg +ve mothers from 75% to 90% did not increase the expected utilities for the screening strategies. | **Key findings:**  Currently mass HBV vaccination the least ‘cost-effective’ of the strategies, however a reduction in price may change this finding.  **Study strengths (author reported):**  Nil reported.  **Study limitations (author reported):**  Outcome used to calculate the ‘utility’ is protection from HBsAg carrier state, not sequelae of CHB.  Analysis considers vertical, not horizontal HBV transmission (except reducing utility from ‘no vaccination’ in HBe-Ag negative mothers to 0.9 rather than 1).  Use of data from the Philippines may limit generalisability of findings.  **Study conclusions:**  See ‘key findings’ above. | **Funding:**  U.S. National Academy of Sciences/National Research Council via USAID grant.  **Conflicts of interest:**  Not reported. |

* No separate methods and results sections, presented in the section of the article entitled, ‘a cost-effectiveness analysis’.

| **Title & Abstract** | **Introduction** | **Methods** | **Results** | **Discussion** | **Other** |
| --- | --- | --- | --- | --- | --- |
| Nayagam *et al.* (2016) (35)  **Identification in title as an economic evaluation?**  Cost-effectiveness analysis  **Abstract with clear sections for objectives, perspective, setting, methods, results and conclusions?**  Abstract is clear.  Objectives: examine the cost-effectiveness of early treatment and screening in the community  Perspective: Health provider perspective was used for assessing costs  Setting: The Gambia, at community level  Methods: Markov state transition model  Results: ICER of $540/DALY averted; $645/LY saved;  $511/QUALY gained  Conclusions: Screening and treatment for HBV is likely to be cost-effective intervention | **Statement addressing context for the study?**   - In West Africa, more than 70% of cases of hepatocellular carcinoma are caused by HBV in people younger than 50. - Screening and treatment availability major to reduce HBV-related morbidity and mortality. - Treatment for chronic HBV shown cost affective when no screening in high risk groups in high income countries - The antiviral drug is available at generic price, that makes screening and treatment HBV are potentially feasible - No economic evaluation available for community screening and treatment in low income countries   **Study question:**  What is the cost effectiveness of community screening and treatment strategy for chronic HBV infection in The Gambia?  **Relevance to health policy:**  Provide evidence for resource allocation and inform health policy decision making regarding early diagnosis and treatment of people with asymptomatic HBV infection in sub-Saharan Africa | **Population & subgroups:**  Individuals aged 30 and more, positive to hepatitis B surface antigens in the community  In this group, patients showing liver fibrosis were provided with treatment and monitoring  Cohort used for the model: Age: 38+; Assumption of no resistance to tenofovir, no HBV vaccination,  Baseline burden was used to reflect current practice.  **Setting:**  Western Gambia  **Perspective:**  Health provider perspective  **Interventions compared:**  Screening and treatment  vs. Current practice: No publicly provided screening or treatment at the baseline  **Time Horizon:**  40 Years  **Discount rate:**  3% per year  **Outcome measures:**   - Cost / DALY averted - Cost / QALY gained - Cost/ LY saved.   **Modeling or study-based design:**   - Modeling - Decision tree combined with Markov models   **Source(s) for resource use:**   - Literature review - Literature search - PROLIFICA data, public health facility activity data interviews of the health personnel, WHO-CHOICE data - Mean cross-country health utilities by levy et al.   **Date(s) of resource use, currency, year:**   - Costs are expressed in 2013 US$ - Disability weights used from the Global Burden of Disease Study 2010, and approximated from other liver diseases weights if data not available   **Methods for analysis described?**   - Decision tree combined with Markov Models representing the untreated and treated natural history of chronic HBV infection. - 8 Health domains were defined. - Simulation of the disease progression in the cohort in annual cycles over years. - ICER calculation. - Intervention estimated cost-effective if ICER<WTP threshold (range of WTP used here) - Sensitivity analysis performed (one way deterministic and multivariate probabilistic, using second order Monte Carlo Simulations.) - Gamma distribution for costs; beta distribution for probabilities | **Values, ranges, references used reported?**  Cohort: 38 years old +;  The adult HBsAg prevalence is 8.8%; and cohort of 8170 people  **Results of main analysis:**   - Total health benefits for a round of screening vs no screening:   498 additional DALYs averted, 417 LY gained, or  526 QALYs saved.   - The screen and treat intervention vs current practice:   ICERs of $540 / DALY averted  $645 /LY saved  $511 / QALY gained   - Likely to be cost-effective if 3*GDP used as WTP threshold - Not cost-effective if use the WB WTP threshold of $240/ DALY averted   **Sensitivity analysis/es results:**  One way sensitivity analyses:   - ICER remained below three time GDS per capita per DALY averted. - Parameters having a major impact on ICER: HBsAg prevalence, age of the cohort, cost of community screening per person, the price of tenofovir, treatment adherence, screening uptake, rate of progression from BHeAg-neg to chronic HBV infection, and from inactive carrier to HBeAg-neg chronic HBV inf, compensated cirrhosis to hepatocellular carcinoma, health utility calculation for QALYs and discount rate. - Parameters having a small impact: Treatment adherence, screening uptake, varying linkage to care. The following have a minimal impact: in the ICER: resistance to treatment, failure of reduction in disease progression on antiviral treatments, and the annual risk of development of hepatocellular carcinoma for patients with cirrhosis on antiviral therapies   For 2000 Monte-Carlo simulations:   - Mean cost: $44,70 (95% CI 44,39–45) for the screen and treat intervention / $12,45 (95% CI 12–12,91) for current practice - Mean DALYs: 4,215 (95% CI 4,213–4,217) for the screen and treat intervention/4,27 (95% CI 4,268–4,272) for current practice - Mean ICER: $621 (95% CI 612,8–629,6) per DALY averted - WTP threshold of $1460 per additional DALY averted, 99.7% probability of cost effectiveness of the intervention - The probability reduce to 95%, 20% and <1% if WTP threshold is $974, $487 or $240 respectively. - The modelled HBV-negative, untreated HBV-positive, and treated HBV-positive cohorts had median survival ages of 70 years, 62 years, and 69 years, respectively, restoring a nearly normal life expectancy. | **Key findings:**  Screening and treatment of HBV in The Gambia has ICERs of $540/DALY averted, $645 per LY saved and $511 per QALY gained compared to current practice, remaining below the commonly used benchmark WTP threshold of less than three times the country’s GDP per capita  WTP definition crucial in the cost-effectiveness assessment  **Study strengths (author reported):**   - First study to assess cost-effectiveness of active population-level screening and treatment for HBV in LMIC setting - Model incorporated all stages of chronic HBV - Strong sensitivity analysis - Intervention remains cost-effective even with low prevalence of HBV.   **Study limitations (author reported):**   - Cost likely to be overestimated due to field team costs used only for HBV, shifting the setting from ‘real life’ - Cost of community-based HBV screening falls at the lower end of the broad range of community-based HIV screening costs in sub-Saharan Africa presented in a systematic review by Suthar and colleagues - Use of Tenofovir price for costing. Could use Entecavir, cheaper. Pharmaceutical brand is more expensive, and would decrease the cost effectiveness. - Overestimation of linkage to care in the study compared to routine practice. Sensitivity analysis showed low effect of this parameter on ICER - The model underestimates the impact of cot-effectiveness of treatment due to the assumption of static cohort and homogeneous population rather than a dynamic transmission model. Difference estimated limited though. - Health provider perspective excludes household costs, end of life costs. A societal perspective would provide a higher cost-effectiveness. - Generalisability of results possible, but should be taken with precaution - Theoretical WTP and the type of funding of the intervention should be taken into account depending on context before implementing intervention at the national level.   **Study conclusions:**  The community-based screening and treatment for chronic HBV infection is likely to be cost effective if generic-priced tenofovir is used.  Integration of HBV screening with screening for other diseases, using the already established infrastructure for addressing HIV in sub-Saharan Africa, as well as simplifying diagnostic assessment and monitoring, might make this an even more cost-effective intervention.  The combination of vaccination, screening, and treatment raises the possibility of advancing the date of elimination of HBV related morbidity and mortality as a public health threat | **Funding:**  European Commission (FP7)  UK Medical Research Council (MRC)  and the UK Department for International Development (DFID) (under the MRC/DFID Concordat agreement)  **Conflicts of interest:**  MT has accepted fees for advisory boards and lectures from AbbVie,  BMS, Gilead, Janssen, and Merck  All other authors declare no competing interests. |

| **Title & Abstract** | **Introduction** | **Methods** | **Results** | **Discussion** | **Other** |
| --- | --- | --- | --- | --- | --- |
| Vimolket and Poovorawan (2005) (36)  **Identification in title as an economic evaluation?**  Yes, ‘An Economic Evaluation…’.  **Abstract with clear sections for objectives, perspective, setting, methods, results and conclusions?**  Not separated into clear sections. All aspects except for perspective are included in the abstract. | **Statement addressing context for the study?**  “This study was carried out to estimate the economic rationale for introducing routine prenatal HBsAg screening and prescribing combined passive-active immunisation to babies at risk”.  **Study question:**  No question, though objective is clear.  **Relevance to health policy:**  “However, cost-effectiveness studies of maternal HBsAg screening and the vaccination of babies at risk have never been undertaken in the context of health care service in Thailand”. No clear target audience specified. | **Population & subgroups:** Pregnant women.  **Setting:** Thailand.  **Perspective:** Health care-provider, “…confined to direct medical costs. Direct non-medical costs…and indirect costs were excluded…”.  **Interventions compared:**   1. Universal vaccination, screening for HBsAg, HBIG added to vaccine if +ve. 2. Universal vaccination, screening for HBsAg, then for HBeAg if +ve. HBIG added to vaccine only if +ve for both markers. 3. Universal vaccination, no screening. 4. No vaccination, no screening (baseline).   **Time Horizon:** Not specified.  **Discount rate:** No discounting.  **Outcome measures:** Cases prevented.  **Modeling or study-based design:**  Decision tree model, 100 women cohort for incremental analysis, 800,000 women cohort for calculation of total costs. “…a critical review of the literature, with input from HBV experts to ensure accuracy and validity” used to base decision model (unreferenced).  **Preference measures for outcomes:** Not used.  **Source(s) for resource use:**  Data for outpatient hospital care, diagnostic tests and HBIG, from King Chulalongkorn Hospital in Bangkok (average actual patients), costs for HBV vaccine from Disease Control Department (Ministry of Public Health).  **Date(s) of resource use, currency, year:** Cost data for 2004, prices reported in Thai Baht (40 baht = $US 1, no date for conversion).  **Justification for type of decision-analytic model used?** No specific justification for model choice, figure is provided.  **Methods for analysis described?**  Calculation of incremental cost per case prevented (base for each ICER explained in results column). No sensitivity analyses described in the methods. | **Values, ranges, references used reported?**  Values reported in table form, no ranges or references, transition probabilities reported on decision tree diagram rather than in table form.  **Results of main analysis:**   1. Cost = 29,250 baht, expected cases prevented = 99.9, cost-effectiveness (cost/case averted, CE) = 292.79, Incremental cost-effectiveness ratio (ICER) = 95,000 (relative to strategy 2). Total annual cost (for 800,000 cohort) = 234,000,000 baht. 2. Cost = 26,400 baht, expected cases prevented = 99.87, CE = 264.34, ICER = 20,000 (relative to strategy 3). Total annual cost = 211,200,000 baht 3. Cost = 15,000 baht, expected cases prevented = 99.30, CE = 151.05, ICER = 6,521 (relative to strategy 4). Total annual cost = 120,000,000. 4. Cost = 0 baht, expected cases prevented = 97, CE = 0, ICER N/A.   NB. Through strategy 4 cases are still ‘prevented’ (infants are born without HBV infection) because model assumes only 3% of infants will become a carrier via vertical transmission for this population.  **Sensitivity analysis/es results:**  Cost per case prevented decreased for strategy 1 and 2 when costs of screening tests were lowed, though it remained the same for strategy 3. | **Key findings:**  This study provides data to guide screening policy, but there is no clear willingness-to-pay to guide decision-making.  **Study strengths (author reported):**  Nil  **Study limitations (author reported):**  Nil  **Study conclusions:**  Based on current funding, universal vaccination should be continued. With adequate resourcing (double current funding) the authors advocate strategy 2. | **Funding:**  Center of Excellence Research Fund, Chulalongkorn University and Thailand Research Fund.  **Conflicts of interest:**  Not reported. |

| **Title & Abstract** | **Introduction** | **Methods** | **Results** | **Discussion** | **Other** |
| --- | --- | --- | --- | --- | --- |
| Wiwanitkit (2009)* (37)  **Identification in title as an economic evaluation?**  No, ‘cost concern’.  **Abstract with clear sections for objectives, perspective, setting, methods, results and conclusions?**  No abstract. | **Statement addressing context for the study?**  Yes, “Screening for HBV infection in workers pursuing work abroad is a basic practice, and is useful for preventing infected migrating workers from being sent home to their original countries”.  **Study question:**  No specific question  **Relevance to health policy:**  To prevent workers travelling abroad being returned home due to HBV infection.  No clear target audience specified. | **Population & subgroups:** Population travelling abroad for work.  **Setting:** Thailand  **Perspective:** No explicit statement.  **Interventions compared:** Screening before travelling abroad compared to returning home if found to be infected with HBV after travelling abroad for work.  **Time Horizon:** Not specified.  **Discount rate:** No discounting.  **Outcome measures:** Cost incurred for returning home once abroad.  **Modeling or study-based design:**  Hypothetical cohort of 10,000 workers travelling abroad. Likelihood of returning home based on single prevalence trial, via measuring HBsAg.  **Preference measures for outcomes:** Not used.  **Source(s) for resource use:** Cost data for screening not referenced, cost data for returning home sourced from Thai Airways (“current price”).  **Date(s) of resource use, currency, year:** US dollars, no dates provided.  **Justification for type of decision-analytic model used?**  No justification for study design.  **Methods for analysis described?**  Simple cost-comparison (cost-benefit analysis). Range reported considering: a) all workers travelling to countries within Asia and: b) all workers travelling to countries outside Asia, to work. | **Values, ranges, references used reported?**  Values reported for laboratory costs (unreferenced), costs of returning home from within and outside of Asia (no ranges provided).  **Results of main analysis:**  ‘Infectious rate’ of HBV = 4.2%  Cost of screening 10,000 workers = $US 66,666.7  Cost of returning home from within Asia (if no workers screened prior to leaving) = $US 138,666.7  Cost from returning home from outside of Asia (if no workers screened prior to leaving) = $ US 416,000  Cost saving from screening 10,000 workers prior to leaving = $US 72,000 - $US 349,333.3  **Sensitivity analysis/es results:**  Not performed. | **Key findings:**  Screening of working prior to them travelling abroad is likely to be cost saving.  **Study strengths (author reported):**  Nil  **Study limitations (author reported):**  Nil  **Study conclusions:**  “This confirms that the policy for HBV screening is useful, and should be recommended to other settings with similar prevalence of HBV in the population”. | **Funding:**  None.  **Conflicts of interest:**  None. |

* This brief article has no separate sections (e.g. introduction, methods…) and thus data are extracted and placed in the form in the appropriate section.

| **Title & Abstract** | **Introduction** | **Methods** | **Results** | **Discussion** | **Other** |
| --- | --- | --- | --- | --- | --- |
| Zheng et al. (2015) (38)  **Identification in title as an economic evaluation?**  “Economic analysis”  **Abstract with clear sections for objectives, perspective, setting, methods, results and conclusions?**  Clear abstract with identifying objectives, perspective, setting, methods, main results (though results of sensitivity analyses not included), and conclusions. | **Statement addressing context for the study?**  “…the Chinese Government is considering implementing widespread adult HepB vaccination campaigns.  **Study question:**  Aim to perform an economic analysis comparing vaccination +/- screening with an alternative of no vaccination.  **Relevance to health policy:**  Linked to continuing horizontal transmission of HepB in China despite a successful neonatal vaccination program reducing vertical transmission between 1992 and 2006. | **Population & subgroups:**  Describe characteristics of the base case population and subgroups analysed, including why they were chosen.  **Setting:**  Chinese adults aged 21 – 59 years in 2013, using census data. These were analysed as 39 hypothetical cohorts separated into 21 – 39 years and 40 – 59 years.  **Perspective:** analysis considered direct costs (direct medical and direct non-medical) and societal costs (direct cost and indirect costs).  **Interventions compared:**   1. Vaccination with no screening 2. Vaccination with screening of hepatitis B core antigen (anti-HBc) 3. 1) and 2) compared to no vaccination, no screening.   **Time Horizon:**  Costs and consequences considered over a lifetime horizon.  **Discount rate:**  3% discounting rate.  **Outcome measures:**  Costs, in monetary terms, of mortality and morbidity averted through HBV vaccination.  **Modeling or study-based design:**  Model-based study using a decision tree, modelling the likelihood of infection with compared strategies and, if infected, the likelihood of progression through various diseases states (e.g. fulminant disease, death) and costs associated with this. Effectiveness of vaccination at preventing HBV infection estimated as 93% using a previous study.  **Preference measures for outcomes:**  Changes in quality of life were not considered in the model, costs and benefits considered solely in monetary terms.  **Source(s) for resource use:**  Multiple data sources used to estimate costs of compared strategies. These included: published literature (surveillance data, sero-survey data, study data, and expert consensus), several large computerised data sets, Chinese CDC.  **Date(s) of resource use, currency, year:**  All costs considered in 2014 US dollars, with an exchange rate of 6.03 RMB Yuan used. Non-medical costs adjusted to 2014 values using the consumer price index (CPI); medical costs using a medical CPI.  **Justification for type of decision-analytic model used?**  Detailed figure of the model provided, transition probabilities detailed in a separate table. Assumptions of the model described were: 1) nil adverse effects following from HBV vaccination; 2) all vaccinated adults completed a 3-dose course; 3) vaccination confers lifelong immunity and; 4) there was no herd immunity resulting from vaccination (thus a static model was assumed).  **Methods for analysis described?**  Net present value (NPV) and benefit: cost rations (BCRs) to express cost-benefits of the compared strategies. No missing data described, nor were there any half cycle corrections (use of a decision tree disallows formal consideration of a time component by the model). Deterministic sensitivity analyses conducted, with cost variables, transition probabilities, and efficacy varied over well-described ranges. Incidence rates of HBV assumed to be 80% and 120% of the base case results. No probabilistic sensitivity analyses described. No assessment of regional heterogeneity described. | **Values, ranges, references used reported?**  Values use to assess costs and benefits are clearly reported, with appropriate referencing, and ranges described. Rationale for ranges is not clear for all parameters (i.e. distribution type is not specified).  **Results of main analysis:**  Results reported separately for the 21-39 years; 40-59 years; and 21-59 years together.  For 21-39 years:   1. Vaccination without screening: 2. Direct costs: $1.5 billion, Societal costs: $2.1 billion. 3. Direct costs averted: $1.6 billion, Societal costs averted: $3 billion. 4. Direct NPVs: $83.6 million, societal NPVs: $886.4 million. 5. Direct BCR: 1.06, societal BCR: 1.42.   For 40-59 years:   1. Direct costs: $1.4 billion, Societal costs: $2 billion. 2. Direct costs averted: $0.8 billion, Societal costs averted: $1.2 billion. 3. Direct NPVs: -$594.8 million, societal NPVs: - $846.9 million. 4. Direct BCR: 0.59, societal BCR: 0.59   For 21-59 years:   1. Direct costs: $2.9 billion, Societal costs: $4.1 billion. 2. Direct costs averted: $2.4 billion, Societal costs averted: $4.1 billion. 3. Direct NPVs: -$511.2 million, societal NPVs: $39.5 million. 4. Direct BCR: 0.82, societal BCR: 1.01. 5. Vaccination with screening for anti-HBc:   For 21-39 years:   1. Vaccination without screening: 2. Direct costs: $1.6 billion, Societal costs: $2.1 billion. 3. Direct costs averted: $1.9 billion, Societal costs averted: $ 3.6 billion. 4. Direct NPVs: $ 307.8 million, societal NPVs: $ 1,531.9 million. 5. Direct BCR: 1.19, societal BCR: 1.73.   For 40-59 years:   1. Direct costs: $1.5 billion, Societal costs: $2.0 billion. 2. Direct costs averted: $1.0 billion, Societal costs averted: $1.5 billion. 3. Direct NPVs: -483.3 million, societal NPVs: $-528.5 million. 4. Direct BCR: 0.68, societal BCR: 0.73.   For 21-59 years:   1. Direct costs: $3.1 billion, Societal costs: $4.1 billion. 2. Direct costs averted: $2.9 billion, Societal costs averted: $5.1billion. 3. Direct NPVs: $-175.5 million, societal NPVs: $1,003.4 million. 4. Direct BCR: 0.94, societal BCR: 1.25.   **Sensitivity analysis/es results:**  For screening strategy, BCR fell below 1 when transition probabilities were at the lower bound, otherwise remained above 1. In addition to this, for no screening with vaccination the BCR fell below 1 when cost of health outcomes were at the lower bound, and when the risk of developing chronic hepatitis was at the lower bound. | **Key findings:**   - Recommend adult vaccination, for younger adults, accompanied by screening. - This could serve as a model for future programs. - Recommend small pilot to test strategy prior to wider implementation.   **Study strengths (author reported):**   - Uses up-to-date national sero-survey and surveillance data. - Differences based on different geographical regions accounted for in the model. - Followed guidelines from the World Health Organization and US Centers for Disease Control.   **Study limitations (author reported):**   - Costs saving estimates are conservative. - Significant variation across regions may be inappropriate to consider results as nationally representative.     **Study conclusions:**  “…the results of our study support implementation of a HepB vaccination program for young adults. A screening-based vaccination strategy will result in a greater value to society than a vaccination without screening strategy”. | **Funding:**  Funded by the Chinese Ministry of Science and Technology Program for Important Infectious Diseases Control and Prevention.  **Conflicts of interest:**  None declared. |
